# Molecular mechanism of the tree shrew’s insensitivity to spiciness

**DOI:** 10.1371/journal.pbio.2004921

**Published:** 2018-07-12

**Authors:** Yalan Han, Bowen Li, Ting-Ting Yin, Cheng Xu, Rose Ombati, Lei Luo, Yujie Xia, Lizhen Xu, Jie Zheng, Yaping Zhang, Fan Yang, Guo-Dong Wang, Shilong Yang, Ren Lai

**Affiliations:** 1 Key Laboratory of Animal Models and Human Disease Mechanisms of Chinese Academy of Sciences/Key Laboratory of bioactive peptides of Yunnan Province, Kunming Institute of Zoology, Kunming, Yunnan, China; 2 University of Chinese Academy of Sciences, Beijing, China; 3 State Key Laboratory of Genetic Resources and Evolution, and Yunnan Laboratory of Molecular Biology of Domestic Animals, Kunming Institute of Zoology, Chinese Academy of Sciences, Kunming, Yunnan, China; 4 Kunming Primate Research Center, Kunming Institute of Zoology, Chinese Academy of Sciences, Kunming, Yunnan, China; 5 Department of Biophysics and Kidney Disease Center, First Affiliated Hospital, Institute of Neuroscience, National Health Commission and Chinese Academy of Medical Sciences Key Laboratory of Medical Neurobiology, Zhejiang University School of Medicine, Hangzhou, Zhejiang Province, China; 6 Department of Physiology and Membrane Biology, University of California, Davis, Davis, California, United States of America; 7 Center for Excellence in Animal Evolution and Genetics, Chinese Academy of Sciences, Kunming, Yunnan, China; Virginia Polytechnic Institute and State University, United States of America

## Abstract

Spicy foods elicit a pungent or hot and painful sensation that repels almost all mammals. Here, we observe that the tree shrew (*Tupaia belangeri chinensis*), which possesses a close relationship with primates and can directly and actively consume spicy plants. Our genomic and functional analyses reveal that a single point mutation in the tree shrew’s transient receptor potential vanilloid type-1 (TRPV1) ion channel (tsV1) lowers its sensitivity to capsaicinoids, which enables the unique feeding behavior of tree shrews with regards to pungent plants. We show that strong selection for this residue in tsV1 might be driven by *Piper boehmeriaefolium*, a spicy plant that geographically overlaps with the tree shrew and produces Cap2, a capsaicin analog, in abundance. We propose that the mutation in tsV1 is a part of evolutionary adaptation that enables the tree shrew to tolerate pungency, thus widening the range of its diet for better survival.

## Author summary

Most mammals cannot tolerate the pungent sensation, such as that evoked by eating chili peppers. Here, we show that unexpectedly, the tree shrew, a mammal closely related to primates, can consume pungent plants. We determined that this tolerance is caused by an amino acid change in the tree shrew’s transient receptor potential vanilloid type-1 (TRPV1) ion channel, which lowers the channel’s sensitivity to capsaicinoids—the substances that make plants spicy. We attribute the strong selection for this amino acid to an adaptation to consuming *Piper boehmeriaefolium*, a spicy plant that geographically overlaps with the tree shrew and produces Cap2, a substance similar to capsaicin, the pungent agent found in chili peppers. Our study suggests an evolutionary and molecular mechanism adopted by the tree shrew to expand its nutritional repertoire.

## Introduction

Many plants contain pungent chemicals that deter animals from consuming them. Particularly, the genus *Capsicum* encompasses 22 wild species and produces a capsaicinoid called capsaicin, which is a pungent substance [1,2]. One of these species, the chili pepper, is a low shrub with capsaicin-containing fruits that are readily accessible to mammals and birds. However, capsaicinoids in these plants repel animals by evoking a sharp and burning sensation through activation of the nociceptor transient receptor potential vanilloid type-1 (TRPV1) ion channel [3–7]. Interestingly, birds are an exception due to two specific point mutations in their TRPV1 channels that render them insensitive to capsaicin [8,9]. This adaptation broadens the range of diet in birds and also confers an advantage to the plants, because their seeds can be widely distributed by the birds [10]. Humans have an acute sensitivity to spicy food, and many find it unbearable. Nonetheless, with training, some have learned to enjoy the burning sensation elicited by consuming spicy food [1], which presumably also confers protection against bacterial and fungal infection in the human digestive system [11]. However, whether pungency tolerance exists in other mammals remains unexplored.

The tree shrew (*Tupaia belangeri chinensis*) is a mammal closely related to primates. Genomic analysis suggests that the tree shrew has a high sequence similarity to human [12]. Surprisingly, we found that tree shrews actively fed on the chili pepper when it was provided to them. To understand the molecular mechanism responsible for this capsaicin insensitivity, we employed a combination of behavioral observation, genome scans, mutational analyses, and electrophysiology studies, and we also sought a plausible cause for the behavior change that could not be brought about by a need to consume the chili pepper, a plant that was geographically isolated until recent times. We identified an amino acid change in the tree shrew TRPV1 (tsV1) at the residue that in the homologs of sensitive species forms a hydrogen bond with capsaicinoids and stabilizes the binding, and we show that this change renders tsV1 refractory to capsaicinoid-induced activation. Furthermore, we identified Cap2, a capsaicin analogue, from *Piper boehmeriaefolium* [13], which overlaps geographically with tree shrews. We speculate that Cap2 might act as a potential environmental pressure for positive selection of the molecular change in tsV1 that confers capsaicinoid tolerance.

## Results

### The tree shrew can tolerate the chili pepper

**Fig 1:**
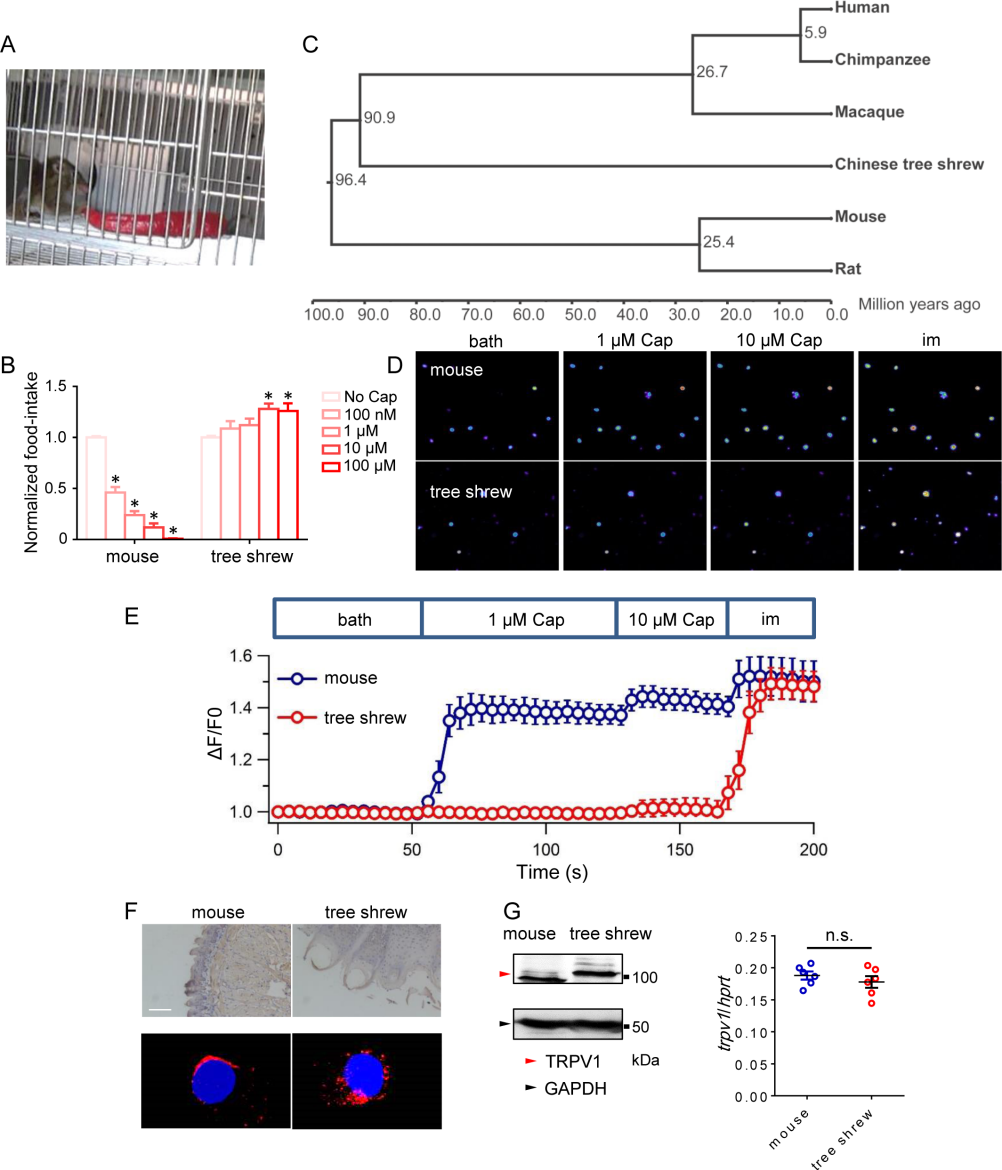
Tree shrews exhibit insensitivity to spiciness. (A) Tree shrew directly consumes chili pepper. (B) The food intake of manufactured diet with different capsaicin concentration. All the values were normalized by the average quantity of manufactured diet without capsaicin (*n* = 3, * p ≤ 0.001). (C) Consensus phylogenetic tree of species used in this study. The divergence time is labeled on the nodes. (D) Calcium imaging of DRG neurons from mice (top row) or tree shrews (bottom row) challenged sequentially with capsaicin (1 μM and 10 μM) and ionomycin (1 mM). (E) Representative calcium fluorescence signals of DRG neurons from mice or tree shrews were counted from representative cells (*n* = 10 per data point). (F) Representative photomicrographs of tongue sections from mice or tree shrews stained with TRPV1 antibody (top) and TRPV1 immunofluorescence staining (in red) of representative DRG neurons from mice or tree shrews (bottom). Nuclear DNA (in blue) was stained with DAPI (scale bar, 100 μm). (G) The expression level of TRPV1 protein (left panel) in mouse and tree shrew DRG. Transcript expression level (right panel) of *trpv1* in mouse and tree shrew DRG. The underlying data of panels B, E, and G can be found in S1 Data. DRG, dorsal root ganglia; TRPV1, transient receptor potential vanilloid type-1

Among mammals, humans are the only known species that deliberately seeks spicy sensation from food [1]. Fortuitously, we observed that, like humans, tree shrews also actively fed on the chili pepper (**Fig 1A** and **S1 Movie**). Moreover, when capsaicin was added to the food, the food intake in mice was significantly reduced in a concentration-dependent manner, whereas we found no such change in tree shrews (**Fig 1B**). These observations suggest that spiciness elicited by capsaicin is tolerated in tree shrews.

To determine whether genomic changes or conditioning is responsible for capsaicin tolerance in tree shrews, we applied a genomic scan for positively selected genes (PSGs) among one-to-one orthologous genes in the tree shrew genome and 5 other phylogenetically closely related mammals: humans, chimpanzees, macaques, mice, and rats. The phylogenic relationship of these 6 species is shown in an established species tree (**Fig 1C**). Three hundred seventy-three PSGs were retained after applying the False Discovery Rate correction to the PSGs (**S2 Data**). Gene ontology (GO) enrichment analysis by Protein Analysis THrough Evolutionary Relationships (PANTHER) Overrepresentation Test of the 373 PSGs identified 71 over-represented GO terms (**S1 Table**). Interestingly, there was a clear enrichment of chemosensory behavior–related genes (GO: 0007635, *P* = 0.0329), including *trpv1*, as being over-represented by PSGs. This observation suggested that spiciness tolerance in tree shrew may be due to a positive selection of *trpv1* in the tree shrew.

### TRPV1^+^ nociceptors from tree shrews are poorly activated by capsaicin

We took advantage of the capsaicin tolerance to probe the effects of positive selection on the tsV1 channel protein. We first analyzed capsaicin-induced activity in dissociated mouse and tree shrew dorsal root ganglia (DRG) neurons by calcium imaging. Consistent with observations in the behavioral tests, we found that tree shrew DRG neurons exhibited a much weaker response to capsaicin compared with mouse DRG neurons (**Fig 1D and 1E**). This was not caused by a paucity of tsV1 expression, because immunohistochemical and quantitative analyses of tissue sections from tongue and DRG suggested similar levels of TRPV1 protein and mRNA between mouse and the tree shrew (**Fig 1F and 1G**). In addition, using hematoxylin and eosin (H&E) staining, we observed that unlike in mice, capsaicin didn’t cause discernible tissue disruption in the tree shrew (**S1A Fig**). Furthermore, capsaicin-induced acute pain behavior was absent in tree shrews, despite a high concentration of capsaicin used (**S1B Fig**). To rule out that the absence of capsaicin-induced acute pain in tree shrews is due to any other defects in the pain sensation pathway, we investigated formalin-induced pain. In mice, local injection of formalin strongly elicited pain (**S1C Fig**). In contrast to the tolerance to capsaicin, formalin elicited both acute and inflammatory pain in the tree shrew which is similar to mice (**S1C Fig**). We also observed numerous inflammatory neutrophils and serious disruption of tissue structure in the formalin-injected hind paw of both mice and the tree shrew (**S1D Fig**). These observations suggest that pain sensation is intact in the tree shrew and are consistent with the hypothesis of a reduced sensitivity of tsV1 to capsaicinoids such as capsaicin.

**Fig 2:**
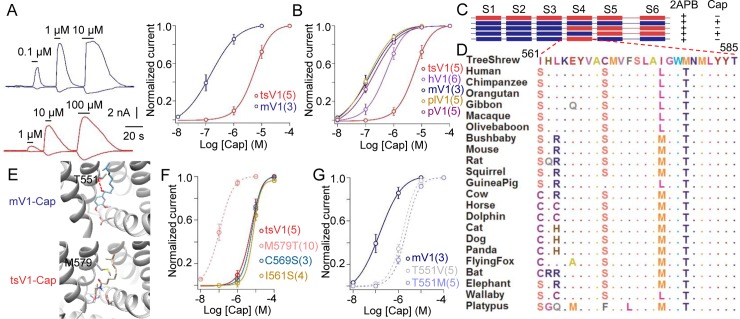
Mutation on site 579 endows tsV1’s tolerance of capsaicin. (A) Representative whole-cell current traces of mV1 (shown in blue) and tsV1 (shown in red) elicited by capsaicin from whole-cell recording at +80 and −80 mV (left panel). Comparison of capsaicin responses of mV1 (blue line) and tsV1 (red line) overlapped with fits of a Hill equation (right panel). (B) Comparison of capsaicin responses of mV1, hV1, tsV1, plV1, and pV1 overlapped with fits of a Hill equation. (C) Responsiveness to capsaicin and 2APB by chimeric channels between mV1 and tsV1. (D) The amino acid sequence representing S3–S4 linker and S4 domain from tsV1 is aligned with the corresponding sequences from other 22 mammals’ TRPV1. (E) A zoomed-in view of capsaicin binding pocket of mV1. A representative configuration of docked capsaicin is shown (upper panel). Docking of capsaicin onto a zoomed-in view of S3–S4 linker and S4 domain of tsV1 (lower panel). (F) Concentration-response curves for tsV1 and channel single-point mutants overlapped with fits of a Hill equation. (G) Comparison of capsaicin responses of mV1 (blue solid line), mV1_T551V (gray dash line), and mV1_T551M (blue dash line) overlapped with fits of a Hill equation. The number of the tested cells is indicated. All values are given as average ± s.e.m. The underlying data of panels A, B, F, and G can be found in S1 Data. 2APB, 2-aminoethoxydiphenyl borate; hV1, human TRPV1; mV, mini volt; mV1, mouse TRPV1; plV1, platypus TRPV1; pV1, polar bear TRPV1; TRPV1, transient receptor potential vanilloid type-1; tsV1, tree shrew TRPV1

To directly measure the capsaicin sensitivity of tsV1, we expressed tsV1 in mammalian cells and performed whole-cell recording (**S2A Fig** and **Fig 2A**). We observed that the concentration of capsaicin required to activate tsV1 (EC_50_: 5.2 ± 0.13 μM, *n* = 5) is much higher than the concentrations required to activate mouse TRPV1 (mV1) (EC_50_: 0.2 ± 0.07 μM, *n* = 3) (**Fig 2A**) and other mammalian TRPV1 channels we tested (**Fig 2B**). In contrast, tsV1 showed similar single-channel conductance and similar sensitivity to low pH and heat as the other mammalian TRPV1 channels (**S2B**–**S2D Fig and S3 Fig**). Based on these results, we reasoned that capsaicin tolerance we observed was caused by the reduced tsV1 sensitivity to capsaicin.

### A single conserved amino acid is responsible for reduced capsaicin sensitivity in tsV1

To understand the reduced sensitivity in tsV1, we first examined the protein sequence of the channel. Significant positive selection signal was detected in tsV1, and Bayes Empirical Bayes (BEB) testing identified 30 sites under positive selection (**Table 1** and **S2 Table**). Since these 30 sites are widely distributed in different structural elements of tsV1, we constructed chimeric channels between tsV1 and mV1 to identify a region or regions in tsV1 that could cause a reduced binding affinity to capsaicin and thus result in capsaicin tolerance (**Fig 2C**). We found that a swap of the transmembrane domain IV (S4) including S3–S4 linker had a major impact on capsaicin sensitivity, whereas changing other domains did not have a significant effect (**Fig 2C**). Based on a homology model of tsV1 (**S4A Fig**), we found that among the 30 positively selected sites, three (I561, C569, and M579) are located within the S4 domain and the S3–S4 linker, which are close to the capsaicin-binding pocket (**Fig 2D**), suggesting that these residues are more likely to be responsible for the reduced capsaicin sensitivity. To test the functional properties of these three sites, we replaced them with the homologous residues in mV1 and compared their sensitivity to capsaicin with wild type tsV1 by whole-cell recording. M579 in tsV1 corresponds to T551 in mV1, a site forming a hydrogen bond (**Fig 2E upper panel**, dotted line in red) with capsaicin that stabilizes the binding [14–16], whereas M579 in tsV1 cannot make a hydrogen bond with capsaicin (**Fig 2E lower panel**). When we replaced the methionine with a threonine (M579T), which can form the hydrogen bond with capsaicin, the mutant became extremely sensitive to capsaicin with an EC_50_ value of 0.1 ± 0.26 μM, which is approximately 50-fold lower than the EC_50_ of tsV1 (**Fig 2F**). Conversely, mutant mV1 with T551 replaced by a methionine or a valine which cannot form a hydrogen bond with capsaicin, capsaicin sensitivity was significantly reduced to about 10% of the wild type mV1 (mV1_T551M, EC_50_ approximately 2.1 μM; mV1_T551V, EC_50_ approximately 1.5 μM, and wild-type mV1, EC_50_ approximately 0.2 μM) (**Fig 2G**). These results suggest that the observed decrease in capsaicin sensitivity is caused by a structural change that reduces the binding affinity between tsV1 and capsaicin.

**Table 1:** Positive selection on tree shrew TRPV1.

*N*^#^	lnL0	lnL1	2ΔlnL	*P* value	omega	Positive selection sites
**6**	**−5816.62**	−**5809.48**	14.28**	**1.58E**^**−4**^	**7.28**	30H, 35T*, 42R, 44P, 48R, 55E, 57G, 75P, 92S, 95D, 100C, 101R, 113L, 114K, 130T, 144K, 145K*, 152E, 166K, 309K, 561I, 569C, 579M, 632D*, 633R*, 635R*, 636T*, 637S, 642M, 722M

# number of sequences

* present 5% significant level

** present 1% significant level.

**Abbreviation:** lnL, log likelihood; TRPV1, transient receptor potential vanilloid type-1.

### Tree shrews tolerate a local *Piper* species containing capsaicinoid similar to capsaicin

We next explored a plausible environmental factor that could select for the structural change in tsV1 and behavioral change in the species. By sequencing TRPV1 fragments containing the 579 site in 155 wild tree shrews from 5 populations (Genbank No. MF073026–MF073180), we found that M579 is conserved in all sequenced individuals (**S4D Fig**), suggesting that it has been fixed at the species level. This excludes the chili pepper, which had been geographically isolated from the tree shrew until it was introduced to South Asia only approximately 300 years ago [2].

**Fig 3:**
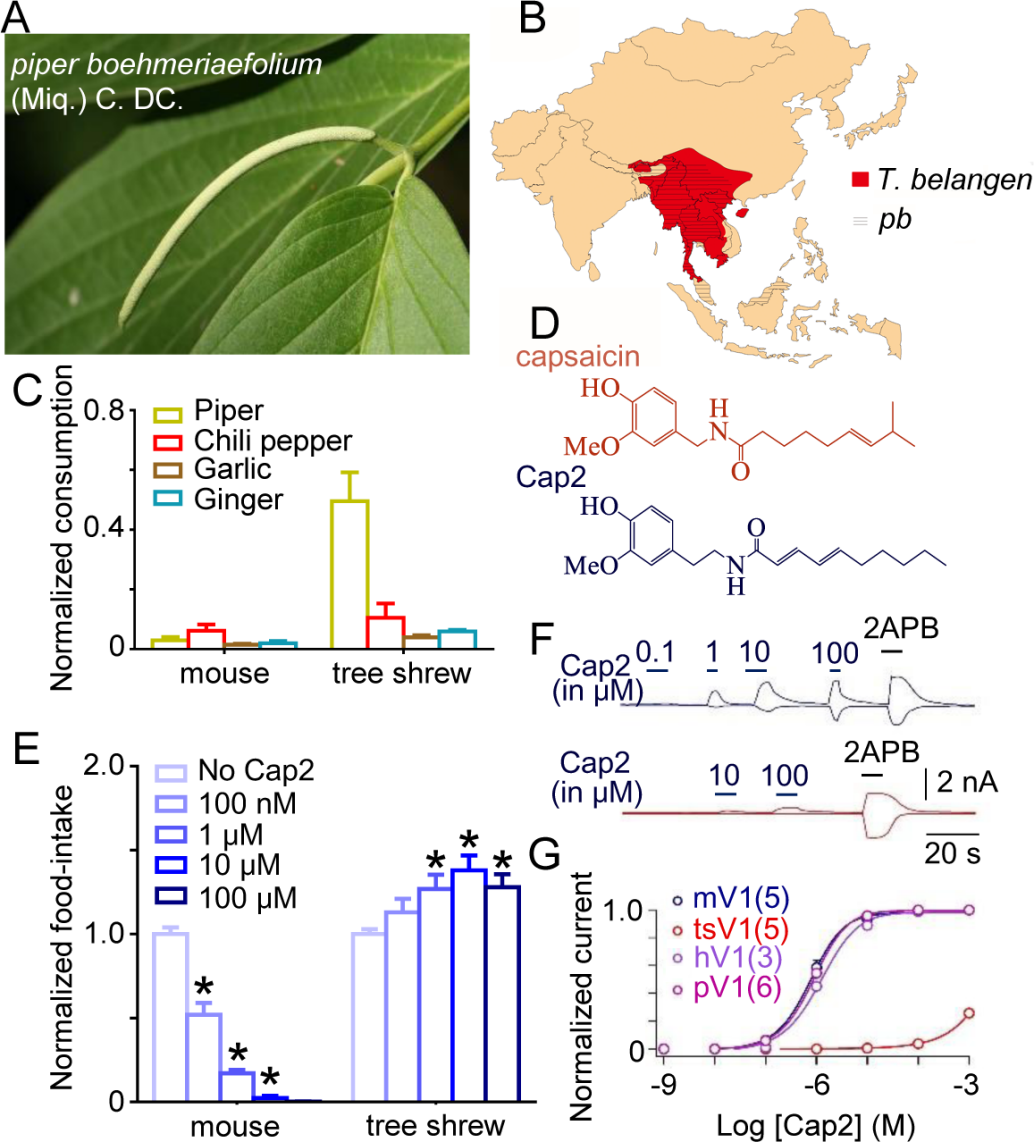
Tree shrew and tsV1 show insensitivity to Cap2, a TRPV1 agonist, from *Piper* species. (A) Image of *Piper boehmeriaefolium* (Miq.) C. DC. (Piperaceae). (B) Map of South Asia showing the distribution of *Tupaia belangeri chinensis* and *P*. *boehmeriaefolium*. (C) Comparison of food consumption (apple, garlic, ginger, *P*. *boehmeriaefolium*) over 48 hours. The food consumption of wild mice (*Niviventer confucianus*) and wild tree shrews were normalized by apple consumption (*n* = 3, p ≤ 0.001). (D) Structural comparison of capsaicin (in red) and Cap2 (in blue). (E) The food intake of manufactured diet with different Cap2 concentration. All the values were normalized by the average weight of manufactured diet without Cap2 (*n* = 3, *p* ≤ 0.001). (F) Representative current traces of mV1 (blue line) and tsV1 (red line) from whole-cell recording at +80 and −80 mV. (G) The comparison of dose-response relationships for Cap2 among the tree shrew (EC_50_ = 1.9 ± 0.03 mM, *n* = 5), human (EC_50_ = 5.73 ± 0.05 μM, *n* = 3), mouse (EC_50_ = 0.74 ± 0.05 μM, *n* = 5), and polar bear (EC_50_ = 0.87 ± 0.04 μM, *n* = 6) TRPV1 channels. The number of the tested cells is indicated. All values are given as average ± s.e.m. The underlying data of panels C, E, and G can be found in S1 Data. mV, mini volt; mV1, mouse TRPV1; TRPV1, transient receptor potential vanilloid type-1; tsV1, tree shrew TRPV1

*Piper boehmeriaefolium* (Miq.) C. DC., a spicy *Piper* species, has a geographical distribution that overlaps that of the tree shrew [17] (**Fig 3A and 3B**). We therefore investigated if the chemosensory behavior of the tree shrew is *P*. *boehmeriaefolium*-related. Video observation (48 hours in total) revealed that wild tree shrews preferred *P*. *boehmeriaefolium* over other pungent plants, which was different from wild mice (**Fig 3C**). Phytochemical investigation illustrated that *P*. *boehmeriaefolium* is spicy because it contains a chemical analog of capsaicin (**Fig 3D**, we referred to Cap2) [13], which possesses an additional carbon in the “neck” region of capsaicin as well as several small differences in the “tail” region (**Fig 3D**). Therefore, we hypothesized that the changes in tsV1 have been evolutionarily selected by Cap2 in the *Piper* species.

To test this hypothesis, we chemically synthesized Cap2 (**S4B** and **S4C Fig**) and added it into the food of tree shrews and mice in different concentrations. We observed that food intake in mice was significantly decreased with the increase in Cap2 concentration; in contrast, increasing Cap2 did not reduce but caused a slight increase in the food intake in tree shrews (**Fig 3E**). In agreement with the food intake observation, we found that in whole-cell recordings tsV1 exhibited an EC_50_ of approximately 1.9 ± 0.03 mM, *n* = 5, which was approximately 2,500-fold larger than the EC_50_ of mV1 (0.74 ± 0.05 μM, *n* = 5) and other mammalian TRPV1 (**Fig 3F and 3G**). These results suggest that ability to feed on *P*. *boehmeriaefolium* may be the driver for the spread of tsV1 mutation in the tree shrew.

### Cap2 is a potential environmental driver causing M579 fixation in tsV1

**Fig 4:**
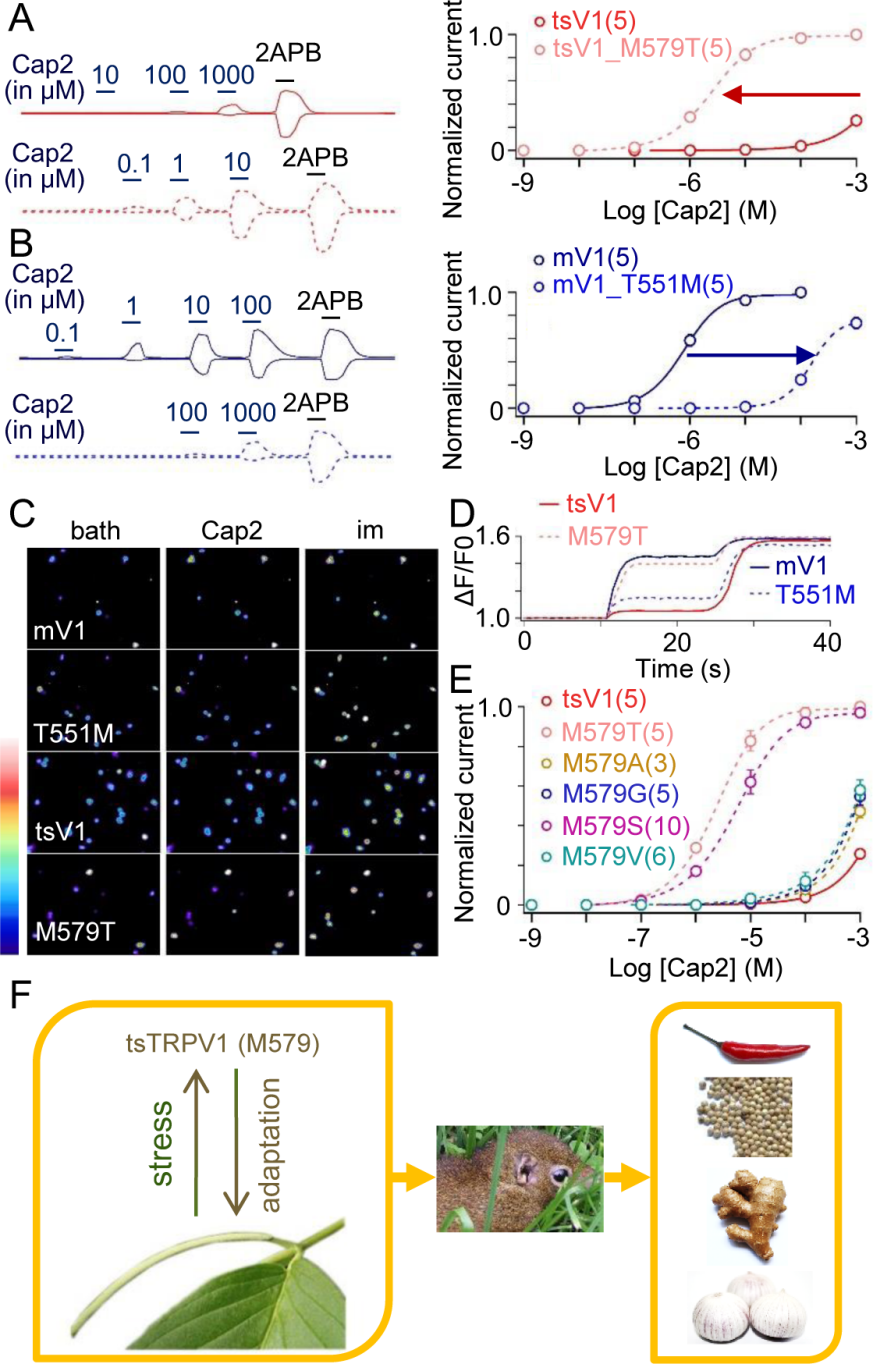
The strong selection on site 579 is due to *Piper boehmeriaefolium*. (A) Comparison of Cap2 responses of tsV1 (solid line) and tsV1_M579T (dashed line). The holding potential was 0 mV, and test potential was at +80 and −80 mV (left panel). Concentration-response curves for tsV1 and tsV1_M579T overlapped with fits of a Hill equation (right panel). The effector concentrations for half-maximum response (average ± s.e.m) are as follows: for tsV1, 1.9 ± 0.03 mM; for tsV1_M579T, 2.34 ± 0.26 μM. The number of the tested cells is indicated. (B) Representative current traces of mV1 (solid line) and mV1_T551M (dashed line) from whole-cell recording at +80 and −80 mV (left panel). Concentration-response curves for mV1 and mV1_T551M overlapped with fits of a Hill equation (right panel). The effector concentrations for half-maximum response are as follows: for mV1, 0.74 ± 0.05 μM; for mV1_T551M, 151.4 ± 0.12 μM. The number of the tested cells is indicated. (C) Calcium imaging of mV1, tsV1, and mutants-expressing HEK293 cells challenged by Cap2 (10 μM) and ionomycin (1 mM), respectively. Scale bar, 140–2,430 AU. (D) Representative calcium fluorescence signals of mV1, tsV1, and mutants-expressing HEK293 cells were counted from representative cells (*n* = 10 cells per point). (E) Dose-response relationships of tsV1 and mutant channels containing a point replacement in site 579. The EC_50_ values of these mutations in site 579 were as follows: 2.34 μM for tsV1_M579T; 5.46 μM for tsV1_M579S; 0.92 mM for tsV1_M579G; 1.07 mM for tsV1_M579A; and 0.83 mM for tsV1_M579V. The number of the tested cells is indicated. (F) A schematic diagram summarizing the evolutionary stress and adaptation in the tree shrew. All values are given as average ± s.e.m. The underlying data of panels A, B, D, and E can be found in S1 Data. AU, arbitrary unit; HEK293 cells, human embryonic kidney cells 293; mV, mini volt; mV1, mouse TRPV1; tsV1, tree shrew TRPV1

We hypothesized that M579 in tsV1 can also account for the insensitivity to Cap2 because of the structural similarity between capsaicin and Cap2 (**Fig 3D**). Indeed, when we restored the hydrogen bonding capability at M579 by mutating it to a threonine, the tsV1_M579T mutant became significantly more sensitive to Cap2 (**Fig 4A left panel**) with an EC_50_ of 2.34 ± 0.26 μM (*n* = 5), which was approximately 1,000-fold lower than the wild-type tsV1 (**Fig 4A right panel)**. In addition, mutating the homologous site 551 from threonine to methionine in mV1 dramatically reduced the sensitivity to Cap2 by approximately 1,000-fold (**Fig 4B**). mV1_T551M also exhibited largely diminished calcium signal in response to Cap2 (**Fig 4C and 4D**). These observations demonstrate that tsV1 M579 (or mV1 T551) has a major impact on Cap2 sensitivity.

As the hydroxyl group on the side chain of threonine is expected to form a hydrogen bond with Cap2 to stabilize its binding to the tsV1_M579T mutant, just like its interaction with capsaicin on mV1, we next replaced M579 in tsV1 with four other amino acids, in addition to threonine, and compared the EC_50_ of Cap2. None of the five mutants exhibited a concentration-response curve to the right of the wild-type tsV1; the two mutants—M579T and M579S—that can presumably form a hydrogen bond with capsaicin and Cap2 exhibited large shifts to the left (**Fig 4E**). This result suggests that methionine at the 579 has been selected to maximally reduce the sensitivity to Cap2.

There are other chemicals that can also activate TRPV1 and elicit a spiciness sensation like capsaicin and Cap2, such as piperine (from black pepper) and gingerol and shogaol (from ginger) [1]. To test whether these irritants also contribute to fixation of the M579 mutation, we investigated whether M579 is critical for the binding of these irritants. Although mutating this homologous site in both mV1 and tsV1 (mV1_T551M or tsV1_M579T) led to shifts in concentration-response curve of piperine, gingerol, and shogaol (**S5 Fig**), change in the EC_50_ of Cap2 (approximately 1,000-fold, **Fig 4E**) was much larger than other irritants (approximately 10-fold for capsaicin; approximately 2-fold for piperine; approximately 6-fold for gingerol; approximately 10-fold for shogaol). These results are consistent with our hypothesis that Cap2, instead of capsaicin and other irritants, is the main driver for the M579 mutation in tsV1 (**Fig 4F**). This is also consistent with our observation that tree shrews compared to mice preferred *P*. *boehmeriaefolium* over other tested pungent plants (**Fig 3C**) and indicates that the chili pepper is not the driver for the M579 in tsV1 (**Fig 4F**). Our observations together favor the idea that feeding adaptation to *P*. *boehmeriaefolium* rich in Cap2 has positive selected for M579 in tsV1.

## Discussion

In this study, we found that tree shrews can tolerate pungent plants such as the chili pepper and *Piper boehmeriaefolium*, which are avoided by most animals because of the pungent sensation they elicit. We show that M579 in tsV1 has been strongly and positively selected to render this tree shrew homolog of TRPV1 less sensitive to the pungent chemical Cap2 in *P*. *boehmeriaefolium*, a *Piper* species with an overlapping geographical distribution in South Asia, and we suggest a plausible driver for the functional change in tsV1 and the dietary change in the species (Fig 4F).

The TRPV1 channel has been the molecular target for selection in evolution [18,19]. For instance, in camels and squirrels, two point mutations occur in the N-terminal ankyrin-repeat domain of TRPV1 to enhance their heat tolerance [20]. In addition, avian TRPV1 with two specific point mutations shows low sensitivity to capsaicin as well, which is consistent with the observation that birds are favored as vectors for seed dispersal [10]. However, it is difficult to determine the contribution of environmental pressure because of the low degrees of sequence conservation between avian and mammalian TRPV1. We show that the 579 site in tsV1 constitutes a critical molecular determinant; this site is occupied in the tree shrew by a methionine which, unlike the threonine in the TRPV1 of species intolerable to pungency, cannot form a hydrogen bond with capsaicinoid ligands (**Fig 2F** and **Fig 4**).

Our suggestion that *P*. *boehmeriaefolium*, instead of ginger, black pepper, and chili pepper, is the main environmental stress responsible for the positive selection of M579 is supported by the observation that swapping methionine with threonine or vice versa at this position (tsV1_M579T or mV1_T551M) caused significantly more change in sensitivity to Cap2 from *P*. *boehmeriaefolium* (approximately 1,000-fold) than to other irritants, such as piperine, gingerol, and shogaol (**Fig 2F**, **Fig 4A–4D** and **S5 Fig**). The feeding behavior described in **Fig 3C** also supports this interpretation. Furthermore, the cultivation history of chili peppers in South Asia is only approximately 300 years [2], yet M579 has been fixed in the tree shrew (**S4D Fig**), which diverged from humans about 90.0 million years ago (**Fig 1C**). Therefore, feeding adaptation to *P*. *boehmeriaefolium* is the most likely explanation to the fixation of this mutation by positive selection, which enabled an expansion of the tree shrew’s dietary repertoire.

## Materials and methods

### Ethics statement

All the animal experiments were performed in accordance with recommendations in the Guide for the Care and Use of Laboratory Animals of Kunming Institute of Zoology, Chinese Academy of Sciences. Experimental protocols using animals in this study were approved by the Institutional Animal Care and Use Committees at Kunming Institute of Zoology, Chinese Academy of Sciences (approval ID: SMKXLLWYH20120520-01).

### Alignment and trimming of one-to-one orthologous genes

There were 12,767 orthologous genes of humans, chimpanzees, macaques, mice, and rats identified by BioMart in the Ensembl public database. OrthMCL identified 13,333 one-to-one orthologous genes in humans and the tree shrew by using mRNA and protein sequences from Ensembl (Human) and TreeshrewDB (tree shrew) [21]. A total of 10,060 overlapped one-to-one orthologous genes were identified in all 6 species. For genes that have more than one transcript, only the longest transcripts were retained. For each pair of one-to-one orthologous genes, conserved codons among 6 species were extracted by using the Gblocks with default parameters after alignment by Prank [22]. After trimming, conserved well-aligned sequences shorter than 300 bp were discarded.

### PSG detection and GO annotation

All gaps (“-”) and unknown sites (“N”) were removed before positive selection tests by using the CODEML program involved in the PAML [23] package. The topology of the 6 species from Ensembl species tree was used as an input tree in the PAML test. The dN/dS ratios (ω) of the tree shrew branch for each orthologous gene were estimated by using free-ratios model. Orthologous genes with dS summed over all branches of the tree >0.5 were retained. Test 2, by comparing the log likelihood (lnL) of positive detection model (A model) and the corresponding null model with ω_2_ = 1 fixed (A null model), was used to detect PSGs on the tree shrew branch (#1). BEB method was used to identify the sites under positive selection. We conducted the False Discovery Rate (FDR) [24] method for multi-correction for all orthologous genes. Genes with FDR <0.01 were identified as PSGs. For each PSG, matched orthologous human Ensembl gene IDs were used in GO annotation and enrichment tests by using the PANTHER Overrepresentation Test (http://pantherdb.org/). Orthologous genes with dS summed over all branches on the tree >0.5 were used as background gene list. GO term was filtered as the standard: *P* value of GO term is smaller than 0.05, at least two PSGs involved in one GO, PSGs involved in one GO term is more than the expected genes (+), fold enrichment is greater than or equal to 2. Only the first-level GO terms were retained.

### Substitution conserved across mammals and impact prediction

Longest CDS frames of *trpv1* from another 17 mammal species were obtained from Ensembl and aligned by MEGA [25] (see S2 Table). Polyphen-2 [26] was used to predict possible impact of amino acid substitutions from human to the tree shrew of 30 BEB sites detected in tree shrew TRPV1.

### Collection of *Piper* species and wild animals

*Piper boehmeriaefolium* (Miq.) C. DC. (Piperaceae) was collected and identified by Dr. Tharanga Aluthwattha from Xishuangbanna Tropical Botanic Garden, Yunnan Province, People’s Republic of China.

Wild mice (*Niviventer confucianus*) were identified and provided by Dr. Quan Li from State Key Laboratory of Genetic Resources and Evolution, Kunming Institute of Zoology, Chinese Academy of Sciences, Yunnan Province, People’s Republic of China. Wild tree shrews were provided by Kunming Primate Research Center, Chinese Academy of Sciences, Yunnan Province, People’s Republic of China.

### Chemicals

Cap2 was synthesized at Zhengzhou Phtdpeptides Pharmaceutical Technology Co., Ltd. Characterization of Cap2 by NMR and MS matched with prior literature. Capsaicin (Sigma-Aldrich, United States of America) and piperine, gingerol, and shogaol (MedChem Express, USA) were purchased. The purity of all compounds was > 99%.

### Diet preference assays

Wild tree shrews (both sexes, *n* = 5) or mice (both sexes, *n* = 6) were captured. Each animal was maintained in an observation chamber (100 × 100 × 50 cm^3^) at 25–27 °C and allowed free access to food (normal laboratory chow) and tap water ad libitum for at least 5 days before the test. Diet options (apple, *Piper* species, chili pepper, garlic, and ginger) were offered with video recordings. After 48 hours, food intake was quantified.

Different concentrations of vanilloid compound (Cap2 or capsaicin) in manufactured feeds were made as 100 nM, 1 μM, 10 μM, and 100 μM. Along with normally manufactured feeds, these options were offered respectively in food containers for BALB/c mice (both sexes, *n* = 5) or clean tree shrews (both sexes, *n* = 5). After 24 hours, food intake was quantified.

### Molecular biology, cell transfection, and electrophysiology

The tongue cDNA library of tree shrews was constructed by using SMART cDNA

Library Construction Kit (Clontech, USA), as previously reported [27]. A pair of primers (5′-ATGCTGAAGTCTAAGGACGGC-3′ and 5′-CTTCTCCCCTGAAGCCGGGGA-3′) was used to clone tsV1 cDNA from the tongue cDNA library of tree shrews. To promote identification of transfected cells, enhanced green fluorescence protein (eGFP) was genetically linked to the C terminus of tsV1 cDNA by homologous recombination method, as previously described [28]. Single point mutants of TRPV1 were obtained by using Fast Mutagenesis Kit V2, (SBS Genetech Co.,Ltd., China) which were sequenced to confirm that site-directed mutagenesis was made.

HEK293T cells were cultured in Dulbecco's modified eagle medium (DMEM) plus 10% fetal bovine serum with 1% penicillin/streptomycin, incubated at 37 °C in 5% CO_2_. Cells were transiently transfected with cDNA constructs by Lipofectamine 2000 (Life technologies, USA) following manufacturer's protocol. Patch-clamp recordings were performed 1–2 days after transfection.

The macroscopic currents were recorded by employing a HEKA EPC10 amplifier with the PatchMaster software (HEKA). Both pipette solution and bath solution contained 130 mM NaCl, 3 mM HEPES, and 0.2 mM EDTA (pH 7.4). The membrane potential was held at 0 mV, the currents were evoked from +80 mV (500 ms) to −80 mV (500 ms). All recordings were performed at room temperature.

### Heating experiment by laser

The experimental set-up for rapidly heating cell membrane containing expressed TRPV1 channels was described in a previous report [29]. Briefly, a glass pipette was filled with a solution distinct from the bath solution and was centered at the end of the optical fiber. The laser driving power was adjusted to produce junction potential values matching those measured in the same solutions at different temperatures.

### Animal pain model

Capsaicin induced acute pain model was constructed as previously described [28]. Briefly, the right hind paw BALB/c mice or tree shrew was injected with capsaicin (100 nM, 1 μM, 10 μM, and 100 μM). The time spent licking the injected paw by each animal was recorded (within 10 minutes) as soon as the capsaicin was injected.

According to our previous study [30], a formalin-induced inflammatory pain model was established by right hind paw injection of 20 μl 0.5% formalin. A digital video camera was used to revalue the time spent licking the injected paw during phase I (0–5 minutes post-injection) and phase II (15–30 minutes post-injection).

Animals were euthanized via CO_2_ inhalation followed by decapitation. Then, the injected (capsaicin or formalin) right hind paws and tongue tissues were cut off and fixed in 10% formalin solution for H&E stain assay.

### Histological analysis

After fixation by 10% formalin and dehydration by an increasing concentration of alcohol, paw materials were embedded in paraffin and sectioned to a thickness of 5 μm using a histocut (Leica, RM2235, Germany). The same procedure was repeated for tongue tissues of both mouse and tree shrew. Sections of paw tissues were deparaffinized and rehydrated for H&E staining.

Similarly, sections of tongue tissues were also deparaffinized and rehydrated for immunohistochemistry (IHC) analysis. For IHC analysis, sections were incubated with rabbit polyclonal TRPV1 antibodies (Thermo Fisher, USA) and 2% bovine serum albumin (BSA) at 37 °C for 1 hour. After washing in PBS, the sections were exposed to horseradish peroxidase labeled antirabbit IgG (Thermo Fisher, USA) for 1 hour at room temperature. Immunoreactivity was visualized by incubation with 0.05% DAB·4HCl. Stained sections were observed by light microscopy (Olympus, X81, Japan).

### Immunocytochemistry analysis

Mouse or tree shrew DRG neurons or transiently transfected HEK 293 cells were fixed with 4% paraformaldehyde for 10 minutes. Fixed cells were incubated with rabbit polyclonal TRPV1 antibodies (Thermo Fisher, USA) and 2% bovine serum albumin (BSA) at 37 °C for 1 hour. After washing in PBS, the cells were incubated with Cy3 labeled antirabbit IgG secondary antibodies (KPL, USA) and 2% bovine serum albumin (BSA) at 37 °C for 1 hour. One μg/ml DAPI (Roche Diagnostics, Switzerland) was used to dye the nuclear DNA. Cy3 was detected with a main beam splitter at 550 nm and a 570-nm long pass emission filter. DAPI was detected with a main beam splitter at 359 nm and a 570-nm long pass emission filter. DAPI-stained cells were viewed under a confocal laser scanning microscope (Olympus, FV1000, Japan).

### Quantitative real-time PCR and western blot

Total RNA was isolated from DRG by using TRIzol reagent (Invitrogen). cDNA was reverse transcribed from 1 μg of RNA by using M-MLV reverse transcriptase (Promega). Quantitative real-time PCR (qRT-PCR) was performed on the Bio-Rad CFX-96 Touch Real-Time Detection System. Primer sequences are listed below.

- Forward Primers: CCACTGGTGTTGAGACGCC (mouse *trpv1*), CTCATGGACTGATTATGGACAGGAC (mouse *hprt*), GTTTGTCAACGCCAGCTACACCGAC (tree shrew *trpv1*) and AGGACCGAAAGACTTGCTCGCG (tree shrew *hprt*).
- Reverse Primers: TCTGGGTCTTTGAACTCGCTG (mouse *trpv1*), GCAGGTCAGCAAAGAACTTATAGCC (mouse *hprt*), AAGCCAGGCCGCCCTTTGGTTTTC (tree shrew *trpv1*) and CAGTCATAGGAATGGATTGATCGC (tree shrew *hprt*).

Protein extraction from isolated DRG. Western blotting was done as described previously [31], and proteins were detected with appropriate primary (Abcam, UK) and secondary (Cell Signaling Technology, USA) antibodies.

### Calcium imaging

Mouse or tree shrew DRG neurons were acutely dissociated and maintained in a short-term primary culture according to procedures as previously described [28]. DRG neuron cells were loaded with Fluo-4 AM in 2 mM Ca^2+^ Ringer’s solution (140 mM NaCl, 5 mM KCl, 2 mM MgCl_2_, 10 mM glucose, 2 mM CaCl_2_, and 10 mM HEPES, pH 7.4). Using ionomycin (1 mM) as blanking control, fluorescence images of DRG neurons were acquired with an Olympus IX71 microscope with Hamamatsu R2 charge-coupled device camera controlled by the MetaFluor Software (Molecular Devices). Fluo-4 was excited by a LED light source (X-Cite 120LED, Lumen Dynamics) with a 500/20-nm excitation filter, while fluorescence emission was detected with a 535/30-nm emission filter. Fluorescence images were acquired with automated routines written in MetaMorph software (Molecular Devices) and analyzed with Igor Pro (Wavemetrics).

### Homology modeling of tsV1 and docking of capsaicin

tsV1 was modeled based on the cryo-EM structure of rat TRPV1 in capsaicin bound state (PBD ID: 3J5R) [32,33]. Briefly, the amino acid sequences of tree shrew and rat TRPV1 were first aligned by Clustal Omega [34,35]. Then, the homology model of tsV1 was built by the RosettaCM program [36]. Ten thousand models were generated, and the model with the lowest energy was chosen as the final model after being refined with the relax application within the Rosetta program [37].

Capsaicin or Cap2 molecule was docked into the ligand binding pocket of mouse and tree shrew TRPV1 channels as described before [14]. In brief, the RosettaLigand application within the Rosetta program [37,38] was used to perform docking. TRPV1 structures were first relaxed within Rosetta program and then the capsaicin was placed roughly in the center of the ligand binding pocket formed by S3, S4, S4–S5 linker, and S6 segments. Ten thousand models were generated, and the top 10 models with the lowest binding energy were selected. The model with lowest binding energy among the largest cluster of the top 10 models was used as the representative model.

### DNA sample collection and sequencing of *trpv1* gene fragment

For association analysis, blood samples and extracted DNA were collected from 155 wild tree shrews using TIANamp Genomic DNA kit according to the manufacturer’s instructions. The primer was designed (CATGGAGACTGCATGGGCAGAAGGGAGCAG) and sequenced a 1 kb fragment covering the gene region of 579 amino acid site in *trpv1*. The wild tree shrews were provided by Kunming Primate Research Center, Chinese Academy of Sciences.

## Supporting information

S1 Fig The construction, trafficking and function of tsV1 and other mammalian TRPV1. (A) H&E-stained paw tissues demonstrating inflammatory reaction was significantly increased following capsaicin injection in mice but not in tree shrews. (B) Paw-licking behavior of mice and tree shrews following injection of 10 μl capsaicin (10 or 100 μM) or saline. The paw licking in response to capsaicin is compared with saline (*n* = 5 mice for each group; *n* = 8 tree shrews for each group, * *p* < 0.001). (C) Paw-licking behavior during phase I (0–5 minutes post-injection) and phase II (15–30 minutes post-injection) of mice and tree shrews following injection of 10 μl formalin (0.8%, v/v) or saline. Animals increased their paw-licking in response to formalin as compared to saline (*n* = 5 mice for each group; *n* = 8 tree shrews for each group, * *p* < 0.001). (D) H&E-stained paw tissues demonstrating formalin was equally effective in inducing inflammatory reaction in mice and tree shrews. All values are given as average ± s.e.m. The underlying data of panels B and C can be found in S1 Data. H&E, hematoxylin and eosin; TRPV1, transient receptor potential vanilloid type-1; tsV1, tree shrew TRPV1.
(TIF)

S2 Fig The construction, trafficking, and function of tsV1 and other mammalian TRPV1. (A) Plasmid construction for eukaryotic expression of tsV1. (B) TRPV1 immunofluorescence staining (in red) of representative tsV1-, hV1-, and mV1-expressing HEK293 cells. Nuclear DNA (in blue) was stained with DAPI. (C) Representative spontaneous single-channel currents of tsV1, hV1, and mV1 recorded at +80 mV. (D) All-point histograms of single-channel events of tsV1, hV1, and mV1. The superimposed curve represents a fit of a double-Gaussian function. The underlying data of panel D can be found in S1 Data. HEK293 cells, human embryonic kidney cells 293; hV1, human TRPV1; mV1, mouse TRPV1; TRPV1, transient receptor potential vanilloid type-1; tsV1, tree shrew TRPV1.
(TIF)

S3 Fig Low pH–and heat–induced responses of tsV1, hV1, and mV1. (A) Comparison of dose-response curves of low pH for tsV1, hV1, and mV1. (B) Heat-induced responses of tsV1, hV1, and mV1 were normalized by 3 mM 2APB-induced currents. The number of the tested cells is indicated. The underlying data of panels A and B can be found in S1 Data. 2APB, 2-aminoethoxydiphenyl borate; hV1, human TRPV1; mV1, mouse TRPV1; tsV1, tree shrew TRPV1.
(TIF)

S4 Fig tsV1 model construction, Cap2 synthesis, and gene sequencing of tree shrew individuals. (A) Channel model of tsV1 (close state) based on the cryo-EM structure of rTRPV1 (PDB 2PNN). (B) Synthesis route of Cap2. (C) Identification of the purity of synthesized Cap2. (D) Alignment of tree shrew *trpv1* from 155 individuals. TRPV1, transient receptor potential vanilloid type-1; tsV1, tree shrew TRPV1.
(TIF)

S5 Fig Mutation on site 579 and its homologous site on mV1 affect the sensitivity to irritants. Comparison of piperine (A), gingerol (B), and shogaol (C) sensitivities of mV1 (blue solid line), mV1_T551M (blue dashed line), tsV1 (red solid line) and tsV1_M579T (red dashed line). The underlying data of panels A–C can be found in S1 Data. mV1, mouse TRPV1; tsV1, tree shrew TRPV1.
(TIF)

S6 Fig Representative configurations of docked Cap2 in capsaicin binding pocket of mV1 and tsV1. (A) A zoom-in view of capsaicin binding pocket of mV1. A representative configuration of docked Cap2 is shown. (B) Docking of Cap2 onto a zoom-in view of S3-S4 linker and S4 domain of tsV1. mV1, mouse TRPV1; tsV1, tree shrew TRPV1.
(TIF)

S1 Table Functional annotation of PSGs in tree shrew based on PANTHER. Only the GO terms passed the standard (see methods) were shown. Fold enrichment value represents the ratio of PSG number to expected gene number. GO, gene ontology; PANTHER, Protein ANalysis THrough Evolutionary Relationships; PSG, positively selected gene.
(DOC)

S2 Table Impact prediction and conservation across mammals of BEB amino acid sites in tree shrew *trpv1* gene. Mutations with polyphen-2 score value ranging from 0–0.7, 0.7–0.9, and 0.9–1 were predicted to be “benign,” “possibly damaging,” and “probably damaging,” respectively. BEB, Bayes Empirical Bayes; TRPV1, transient receptor potential vanilloid type-1.
(DOC)

S1 Data Contains underlying data for figures. (XLSX)

S2 Data Gene ID of 373 PSGs with FDR values smaller than 0.01. Human Ensemble ID of the one-to-one orthologous PSGs was used here. FDR, False Discovery Rate; PSG, positively selected gene.
(PDF)

S1 Movie Tree shrew can directly feed on red chili pepper. (MP4)

## Abbreviations

2APB: 2-aminoethoxydiphenyl borate
BEB: Bayes Empirical Bayes
DMEM: Dulbecco's modified eagle medium
DRG: dorsal root ganglia
eGFP: enhanced green fluorescence protein
FDR: False Discovery Rate
GO: gene ontology
H&E: hematoxylin and eosin
hV1: human TRPV1
IHC: immunohistochemistry
lnL: log likelihood
mV1: mouse TRPV1
plV1: platypus TRPV1
PANTHER: Protein ANalysis THrough Evolutionary Relationships
PSG: positively selected gene
pV1: polar bear TRPV1
TRPV1: transient receptor potential vanilloid type-1
tsV1: tree shrew TRPV1
