# Supplementary figures and images for: Molecular mechanism of the tree shrew’s insensitivity to spiciness
Source: PLoS Biol. 2018 Jul 12;16(7):e2004921. doi: 10.1371/journal.pbio.2004921 (PMC6042686; doi:10.1371/journal.pbio.2004921)

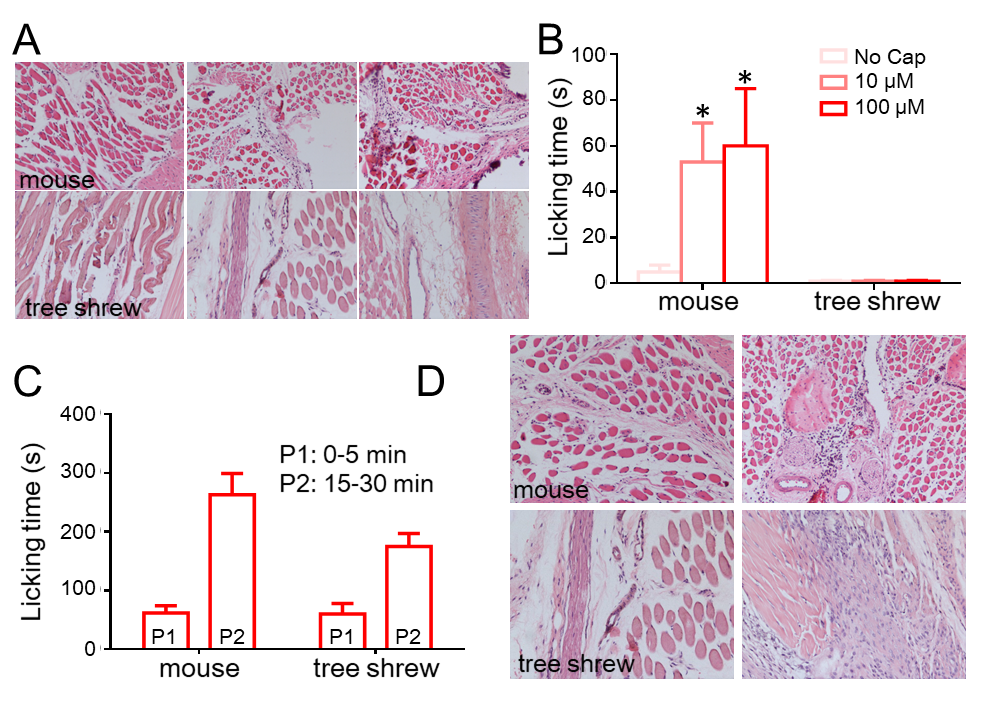

Supplement: S1 Fig — (A) H&E-stained paw tissues demonstrating inflammatory reaction was significantly increased following capsaicin injection in mice but not in tree shrews. (B) Paw-licking behavior of mice and tree shrews following injection of 10 μl capsaicin (10 or 100 μM) or saline. The paw licking in response to capsaicin is compared with saline (n = 5 mice for each group; n = 8 tree shrews for each group, * p < 0.001). (C) Paw-licking behavior during phase I (0–5 minutes post-injection) and phase II (15–30 minutes post-injection) of mice and tree shrews following injection of 10 μl formalin (0.8%, v/v) or saline. Animals increased their paw-licking in response to formalin as compared to saline (n = 5 mice for each group; n = 8 tree shrews for each group, * p < 0.001). (D) H&E-stained paw tissues demonstrating formalin was equally effective in inducing inflammatory reaction in mice and tree shrews. All values are given as average ± s.e.m. The underlying data of panels B and C can be found in S1 Data. H&E, hematoxylin and eosin; TRPV1, transient receptor potential vanilloid type-1; tsV1, tree shrew TRPV1. (TIF) [file pbio.2004921.s001.tif]

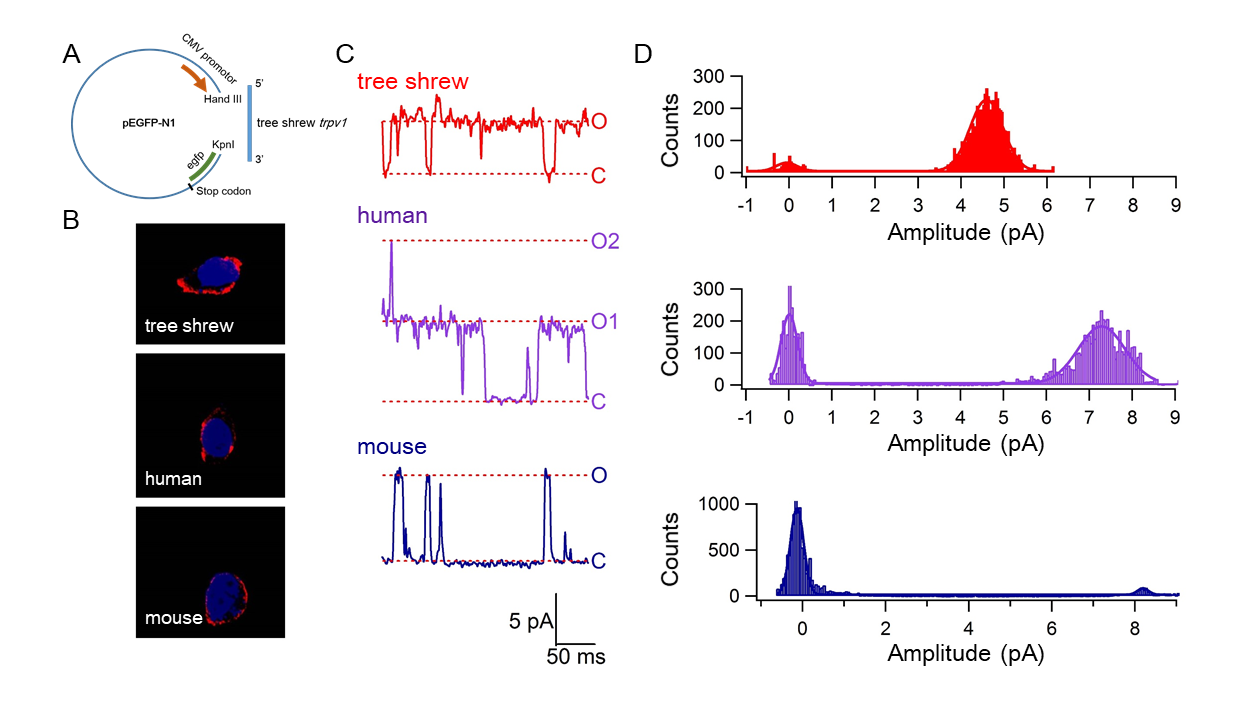

Supplement: S2 Fig — (A) Plasmid construction for eukaryotic expression of tsV1. (B) TRPV1 immunofluorescence staining (in red) of representative tsV1-, hV1-, and mV1-expressing HEK293 cells. Nuclear DNA (in blue) was stained with DAPI. (C) Representative spontaneous single-channel currents of tsV1, hV1, and mV1 recorded at +80 mV. (D) All-point histograms of single-channel events of tsV1, hV1, and mV1. The superimposed curve represents a fit of a double-Gaussian function. The underlying data of panel D can be found in S1 Data. HEK293 cells, human embryonic kidney cells 293; hV1, human TRPV1; mV1, mouse TRPV1; TRPV1, transient receptor potential vanilloid type-1; tsV1, tree shrew TRPV1. (TIF) [file pbio.2004921.s002.tif]

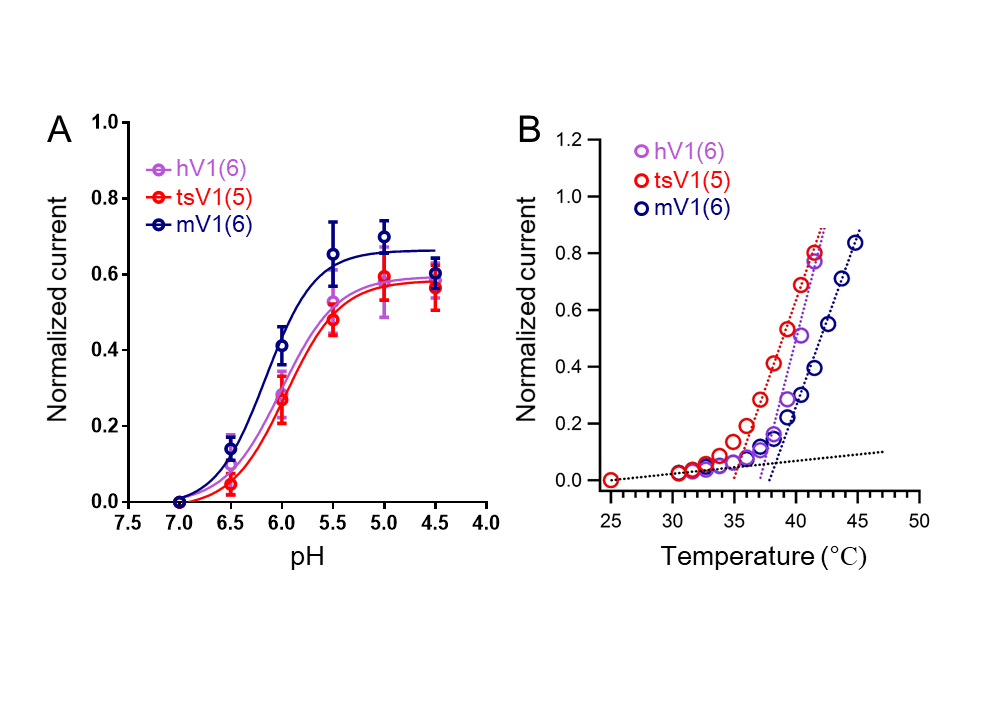

Supplement: S3 Fig — (A) Comparison of dose-response curves of low pH for tsV1, hV1, and mV1. (B) Heat-induced responses of tsV1, hV1, and mV1 were normalized by 3 mM 2APB-induced currents. The number of the tested cells is indicated. The underlying data of panels A and B can be found in S1 Data. 2APB, 2-aminoethoxydiphenyl borate; hV1, human TRPV1; mV1, mouse TRPV1; tsV1, tree shrew TRPV1. (TIF) [file pbio.2004921.s003.tif]

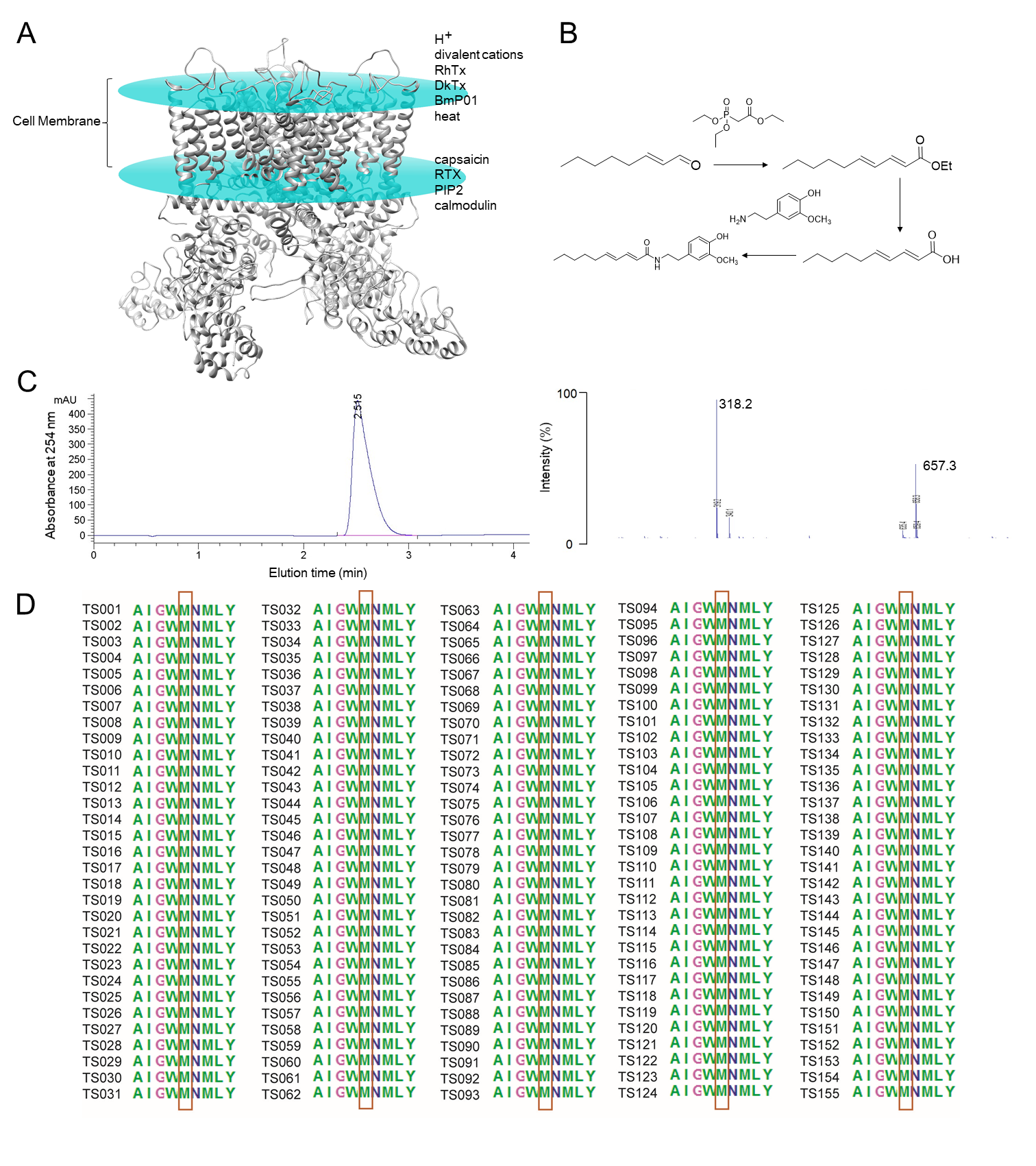

Supplement: S4 Fig — (A) Channel model of tsV1 (close state) based on the cryo-EM structure of rTRPV1 (PDB 2PNN). (B) Synthesis route of Cap2. (C) Identification of the purity of synthesized Cap2. (D) Alignment of tree shrew trpv1 from 155 individuals. TRPV1, transient receptor potential vanilloid type-1; tsV1, tree shrew TRPV1. (TIF) [file pbio.2004921.s004.tif]

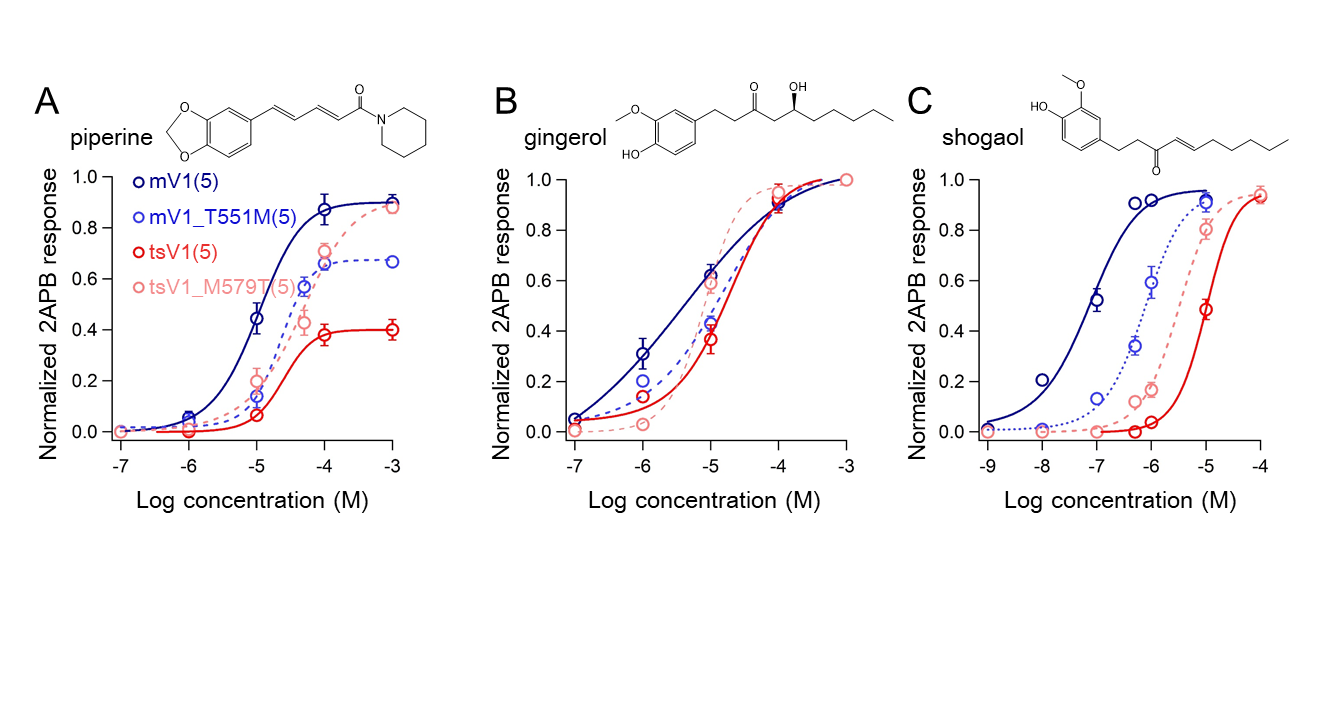

Supplement: S5 Fig — Comparison of piperine (A), gingerol (B), and shogaol (C) sensitivities of mV1 (blue solid line), mV1_T551M (blue dashed line), tsV1 (red solid line) and tsV1_M579T (red dashed line). The underlying data of panels A–C can be found in S1 Data. mV1, mouse TRPV1; tsV1, tree shrew TRPV1. (TIF) [file pbio.2004921.s005.tif]

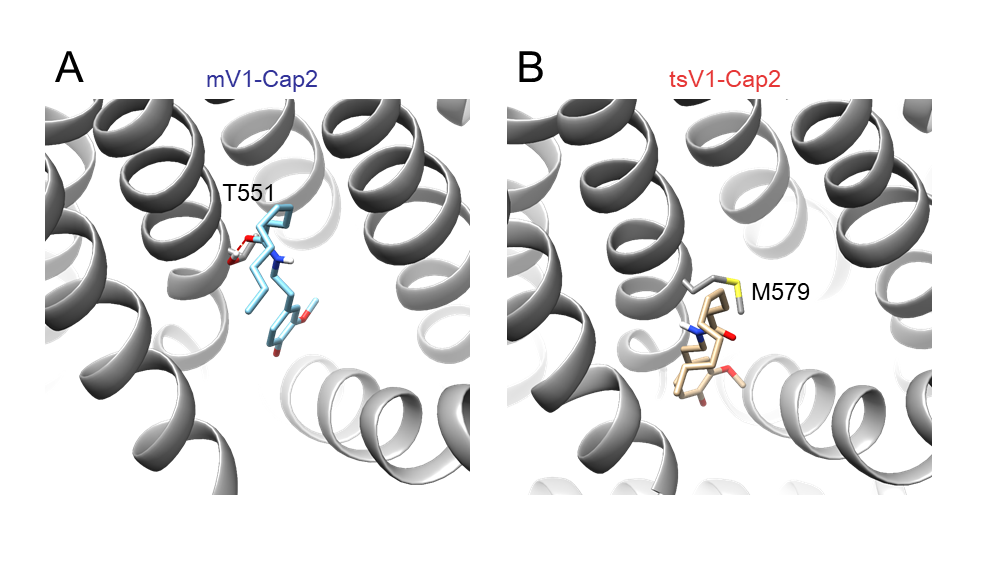

Supplement: S6 Fig — (A) A zoom-in view of capsaicin binding pocket of mV1. A representative configuration of docked Cap2 is shown. (B) Docking of Cap2 onto a zoom-in view of S3-S4 linker and S4 domain of tsV1. mV1, mouse TRPV1; tsV1, tree shrew TRPV1. (TIF) [file pbio.2004921.s006.tif]
