# Supplementary material for: Molecular mechanism of the tree shrew’s insensitivity to spiciness
Source: PLoS Biol. 2018 Jul 12;16(7):e2004921. doi: 10.1371/journal.pbio.2004921 (PMC6042686; doi:10.1371/journal.pbio.2004921)
Supplement: S1 Table — Only the GO terms passed the standard (see methods) were shown. Fold enrichment value represents the ratio of PSG number to expected gene number. GO, gene ontology; PANTHER, Protein ANalysis THrough Evolutionary Relationships; PSG, positively selected gene. (DOC) [file pbio.2004921.s007.doc]

Table S1. Functional annotation of PSGs in tree shrew based on PANTHER.


 

hindgut morphogenesis 	GO:0072757 	4 	2 	0.16	12.42	1.16E-02	
male genitalia morphogenesis 	GO:0007442 	4 	2 	0.16	12.42	1.16E-02	
protein desumoylation 	GO:0048808 	4 	2 	0.16	12.42	1.16E-02	
thymocyte apoptotic process 	GO:0031077 	4 	2 	0.16	12.42	1.16E-02	
post-embryonic camera-type eye development 	GO:0060513 	4 	2 	0.16	12.42	1.16E-02	
cellular response to camptothecin 	GO:0016926 	4 	2 	0.16	12.42	1.16E-02	
regulation of branching involved in salivary gland morphogenesis by mesenchymal-epithelial signaling 	GO:0060665 	4 	2 	
0.16	
12.42	
1.16E-02	
prostatic bud formation 	GO:0070242 	4 	2 	0.16	12.42	1.16E-02	
angiogenesis 	GO:0001525 	174 	14 	7	2	1.21E-02	
collagen catabolic process 	GO:0030574 	22 	4 	0.89	4.52	1.26E-02	
regulation of Ras protein signal transduction 	GO:0046578 	124 	11 	4.99	2.2	1.29E-02	
heart trabecula morphogenesis 	GO:0061384 	23 	4 	0.93	4.32	1.46E-02	
positive regulation of neuroblast proliferation 	GO:0050901 	13 	3 	0.52	5.73	1.61E-02	
leukocyte tethering or rolling 	GO:0002052 	13 	3 	0.52	5.73	1.61E-02	
cellular response to muramyl dipeptide 	GO:0071225 	5 	2 	0.2	9.94	1.77E-02	
type I interferon signaling pathway 	GO:0001954 	25 	4 	1.01	3.97	1.92E-02	
positive regulation of cell-matrix adhesion 	GO:0060337 	25 	4 	1.01	3.97	1.92E-02	
embryonic limb morphogenesis 	GO:0030326 	68 	7 	2.74	2.56	2.15E-02	
production of small RNA involved in gene silencing by RNA 	GO:0040001 	15 	3 	0.6	4.97	2.34E-02	
establishment of mitotic spindle localization 	GO:0050927 	15 	3 	0.6	4.97	2.34E-02	
regulation of histone H3-K9 methylation 	GO:0070918 	15 	3 	0.6	4.97	2.34E-02	
positive regulation of positive chemotaxis 	GO:0051570 	15 	3 	0.6	4.97	2.34E-02	
cellular sodium ion homeostasis 	GO:0072711 	6 	2 	0.24	8.28	2.48E-02	
regulation of miRNA metabolic process 	GO:0006883 	6 	2 	0.24	8.28	2.48E-02	

cytoskeletal anchoring at plasma membrane 	GO:0007016 	6 	2 	0.24	8.28	2.48E-02	
hair follicle maturation 	GO:0048820 	6 	2 	0.24	8.28	2.48E-02	
plasminogen activation 	GO:0035845 	6 	2 	0.24	8.28	2.48E-02	
photoreceptor cell outer segment organization 	GO:0031639 	6 	2 	0.24	8.28	2.48E-02	
positive regulation of dendrite extension 	GO:1903861 	6 	2 	0.24	8.28	2.48E-02	
smoothened signaling pathway involved in dorsal/ventral neural tube patterning 	GO:2000628 	6 	2 	
0.24	
8.28	
2.48E-02	
cellular response to hydroxyurea 	GO:0060831 	6 	2 	0.24	8.28	2.48E-02	
regulation of type I interferon-mediated signaling pathway 	GO:1904837 	16 	3 	0.64	4.66	2.76E-02	
beta-catenin-TCF complex assembly 	GO:0031128 	16 	3 	0.64	4.66	2.76E-02	
developmental induction 	GO:0060338 	16 	3 	0.64	4.66	2.76E-02	
regulation of endothelial cell apoptotic process 	GO:2000351 	29 	4 	1.17	3.43	3.08E-02	
cellular response to interleukin-6 	GO:0071354 	17 	3 	0.68	4.38	3.21E-02	
endothelial cell morphogenesis 	GO:0060561 	7 	2 	0.28	7.1	3.29E-02	
radial glial cell differentiation 	GO:0035584 	7 	2 	0.28	7.1	3.29E-02	
nucleosome disassembly 	GO:0007635 	7 	2 	0.28	7.1	3.29E-02	
regulation of microvillus organization 	GO:0048557 	7 	2 	0.28	7.1	3.29E-02	
embryonic digestive tract morphogenesis 	GO:0001886 	7 	2 	0.28	7.1	3.29E-02	
thyroid gland development 	GO:0006337 	7 	2 	0.28	7.1	3.29E-02	
calcium-mediated signaling using intracellular calcium source 	GO:0060019 	7 	2 	0.28	7.1	3.29E-02	
chemosensory behavior 	GO:0032530 	7 	2 	0.28	7.1	3.29E-02	
apoptotic process involved in morphogenesis 	GO:0030878 	7 	2 	0.28	7.1	3.29E-02	
synaptic vesicle transport 	GO:0048489 	59 	6 	2.38	2.53	3.37E-02	
regulation of smoothened signaling pathway 	GO:0008589 	45 	5 	1.81	2.76	3.69E-02	
complement activation, alternative pathway 	GO:0006957 	8 	2 	0.32	6.21	4.19E-02	

definitive hemopoiesis 	GO:0060216 	8 	2 	0.32	6.21	4.19E-02	
lipoprotein transport 	GO:0042953 	8 	2 	0.32	6.21	4.19E-02	
regulation of myelination 	GO:0055017 	19 	3 	0.76	3.92	4.23E-02	
cardiac muscle tissue growth 	GO:0031641 	19 	3 	0.76	3.92	4.23E-02	
osteoblast differentiation 	GO:0001649 	64 	6 	2.58	2.33	4.67E-02	
B cell receptor signaling pathway 	GO:0050853 	20 	3 	0.81	3.73	4.80E-02	
odontogenesis of dentin-containing tooth 	GO:0042475 	49 	5 	1.97	2.53	4.97E-02	
