# Supplementary material for: Molecular mechanism of the tree shrew’s insensitivity to spiciness
Source: PLoS Biol. 2018 Jul 12;16(7):e2004921. doi: 10.1371/journal.pbio.2004921 (PMC6042686; doi:10.1371/journal.pbio.2004921)
Supplement: S2 Table — Mutations with polyphen-2 score value ranging from 0–0.7, 0.7–0.9, and 0.9–1 were predicted to be “benign,” “possibly damaging,” and “probably damaging,” respectively. BEB, Bayes Empirical Bayes; TRPV1, transient receptor potential vanilloid type-1. (DOC) [file pbio.2004921.s008.doc]

S2 Table. Impact prediction and conservation across mammals of BEB amino acid sites in tree shrew trpv1 gene. Mutations with polyphen-2 score value ranging from 0-0.7. 0.7-0.9 and 0.9-1 predicted to be “benign”, “possibly damage” and “probably damage” respectively.

Human amino acid position	3	8	15	17	21	28	30	47	64	67	72	73	85	86	102	116	117	
Tree Shrew amino acid position	30 	35 	42	44	48	55	57	75	92	95	100	101	113	114	130	144	145	
Polyphen-2 score of amino acid variants from human
to Tree Shrew	0.7	0.145	0.7	0.9	0.2	0.7	0	0	0.2	0.7	0.9	0.9	0.2	0	0.85	0.7	0	
Tree Shrew amino acid 	H 	T 	R 	P 	R 	E 	G 	P 	S 	D 	R 	L 	K 	T 	K 	K 	E 	
Human amino acid 	K 	D 	P 	Q 	C 	D 	N 	T 	P 	E 	C 	Q 	R 	V 	R 	S 	Q 	
Chimpanzee amino acid 	K 	D 	P 	Q 	C 	D 	N 	T 	P 	E 	C 	Q 	R 	V 	R 	S 	Q 	
Orangutan amino acid 	K 	D 	P 	Q 	C 	D 	N 	T 	P 	E 	C 	Q 	R 	V 	R 	S 	Q 	
Gibbon amino acid 	K 	D 	P 	Q 	C 	D 	N 	T 	P 	E 	C 	Q 	R 	V 	R 	S 	Q 	
Macaque amino acid 	K 	D 	P 	Q 	C 	D 	N 	T 	P 	E 	C 	Q 	R 	V 	R 	S 	Q 	
Olivebaboon amino acid 	K 	D 	P 	Q 	C 	D 	N 	T 	P 	E 	C 	Q 	R 	V 	R 	S 	Q 	
Bushbaby amino acid 	K 	D 	L 	Q 	C 	D 	D 	A 	S 	E 	C 	Q 	R 	V 	R 	R 	Q 	
Mouse amino acid 	K 	D 	P 	Q 	C 	D 	N 	T 	P 	E 	C 	Q 	R 	V 	R 	S 	Q 	
Rat amino acid 	Q 	D 	P 	Q 	C 	D 	N 	T 	P 	E 	C 	Q 	R 	V 	R 	S 	Q 	
Squirrel amino acid 	K 	D 	P 	H 	C 	D 	N 	T 	S 	E 	C 	Q 	R 	V 	R 	S 	Q 	
GuineaPig amino acid 	K 	D 	P 	Q 	S 	D 	N 	N 	S 	E 	C 	P 	R 	I 	R 	S 	Q 	
Cow amino acid 	K 	E 	L 	Q 	C 	D 	N 	S 	S 	E 	C 	Q 	R 	A 	R 	K 	Q 	
Horse amino acid 	K 	D 	L 	Q 	C 	D 	D 	S 	S 	E 	C 	Q 	R 	V 	R 	K 	Q 	
Dolphin amino acid 	K 	D 	L 	Q 	C 	D 	N 	S 	S 	E 	C 	Q 	R 	- 	R 	K 	Q 	
Cat amino acid 	K 	D 	P 	Q 	C 	D 	N 	S 	S 	E 	C 	Q 	K 	A 	R 	K 	Q 	

Dog amino acid 	N 	D 	P 	Q 	C 	D 	N 	S 	S 	E 	C 	P 	K 	V 	R 	K 	Q 	
Panda amino acid 	K 	D 	P 	Q 	C 	D 	N 	S 	F 	E 	C 	Q 	K 	V 	R 	K 	Q 	
FlyingFox amino acid 	K 	D 	L 	P 	C 	D 	N 	S 	S 	E 	C 	Q 	K 	V 	R 	K 	Q 	
Bat amino acid 	K 	D 	L 	Q 	H 	D 	N 	S 	S 	E 	C 	Q 	K 	V 	R 	K 	Q 	
Elephant amino acid 	K 	D 	P 	Q 	C 	D 	D 	P 	S 	E 	C 	K 	R 	V 	R 	R 	Q 	
Wallaby amino acid 	K 	E 	P 	- 	C 	A 	G 	S 	H 	E 	Y 	- 	- 	I 	K 	K 	Q 	
Platypus amino acid 	K 	D 	- 	- 	T 	N 	G 	H 	S 	E 	C 	G 	R 	I 	R 	R 	Q 	


Human amino acid position	124	138	281	532	540	550	603	604	606	607	608	613	695	
Tree Shrew amino acid position	152	166	309	561	569	579	632	633	635	636	637	642	722	
Polyphen-2 score of amino acid variants from human to Tree
Shrew	0.56	0	0.13	0.68	0.26	0.879	0.136	0.133	0.852	0	0	0.12	1	
Tree Shrew amino acid 	K	K	K	I	C	M	D	R	R	T	S	M	M	
Human amino acid 	Q	R	Q	S	S	T	K	N	S	L	P	S	K	
Chimpanzee amino acid 	Q	R	Q	S	S	T	K	N	S	L	P	S	K	
Orangutan amino acid 	Q	R	Q	S	S	T	K	N	S	L	P	S	K	
Gibbon amino acid 	Q	R	Q	S	S	T	K	N	S	L	P	S	K	
Macaque amino acid 	Q	R	Q	S	S	T	K	N	S	L	P	S	K	
Olivebaboon amino acid 	Q	R	Q	S	S	T	K	N	S	L	P	S	K	
Bushbaby amino acid 	R	R	R	S	S	T	K	N	S	T	S	T	K	
Mouse amino acid 	Q	R	Q	S	S	T	K	N	S	L	P	P	K	
Rat amino acid 	Q	R	Q	S	S	T	K	N	S	L	P	P	K	
Squirrel amino acid 	Q	R	Q	S	S	T	K	N	S	E	P	S	K	

GuineaPig amino acid 	Q 	R 	Q 	S 	C 	T 	K 	N 	S 	L 	S 	- 	K 	
Cow amino acid 	Q 	R 	Q 	C 	S 	T 	K 	N 	S 	V 	S 	Q 	K 	
Horse amino acid 	Q 	R 	Q 	C 	S 	T 	K 	N 	S 	V 	L 	S 	K 	
Dolphin amino acid 	K 	R 	K 	C 	S 	T 	K 	N 	S 	V 	S 	S 	K 	
Cat amino acid 	Q 	R 	Q 	C 	S 	T 	K 	N 	S 	V 	P 	L 	K 	
Dog amino acid 	Q 	R 	Q 	C 	S 	T 	K 	N 	S 	V 	P 	L 	K 	
Panda amino acid 	Q 	R 	Q 	C 	S 	T 	K 	N 	S 	V 	P 	L 	K 	
FlyingFox amino acid 	Q 	G 	Q 	C 	S 	T 	K 	N 	S 	M 	S 	L 	K 	
Bat amino acid 	Q 	R 	Q 	C 	S 	T 	K 	N 	S 	V 	- 	S 	K 	
Elephant amino acid 	Q 	R 	Q 	S 	S 	T 	N 	K 	P 	A 	A 	S 	K 	
Wallaby amino acid 	H 	K 	H 	S 	S 	T 	Q 	N 	- 	- 	S 	C 	K 	
Platypus amino acid 	L 	R 	L 	S 	F 	T 	K 	N 	T 	E 	C 	Q 	K 	
