# Supplementary material for: Molecular mechanism of the tree shrew’s insensitivity to spiciness
Source: PLoS Biol. 2018 Jul 12;16(7):e2004921. doi: 10.1371/journal.pbio.2004921 (PMC6042686; doi:10.1371/journal.pbio.2004921)
Supplement: S2 Data — Human Ensemble ID of the one-to-one orthologous PSGs was used here. FDR, False Discovery Rate; PSG, positively selected gene. (PDF) [file pbio.2004921.s010.pdf]

Human Ensembl Gene ID	Tree Shrew Protein ID from TreeshrewDB	2lnl	p value	FDR value
ENSG00000153157	TSDBP00000110	15.4548	8.45E-05	0.003037
ENSG00000147419	TSDBP00000170	24.5003	7.43E-07	7.1E-05
ENSG00000163251	TSDBP00000275	17.3671	3.08E-05	0.001407
ENSG00000122591	TSDBP00000364	29.8112	4.76E-08	7.01E-06
ENSG00000132854	TSDBP00000396	30.5307	3.29E-08	5.45E-06
ENSG00000116678	TSDBP00000420	18.1454	2.05E-05	0.001055
ENSG00000047578	TSDBP00000427	29.8562	4.65E-08	7.01E-06
ENSG00000178226	TSDBP00000491	13.3315	0.000261	0.007161
ENSG00000178188	TSDBP00000532	13.3707	0.000256	0.007054
ENSG00000169682	TSDBP00000535	14.5514	0.000136	0.00423
ENSG00000186976	TSDBP00000671	12.745	0.000357	0.00912
ENSG00000115484	TSDBP00000717	16.7298	4.31E-05	0.001825
ENSG00000116981	TSDBP00000908	12.9378	0.000322	0.008377
ENSG00000140326	TSDBP00001010	16.5538	4.73E-05	0.001932
ENSG00000128567	TSDBP00001043	20.7781	5.16E-06	0.000371
ENSG00000196663	TSDBP00001142	23.3314	1.36E-06	0.000122
ENSG00000132016	TSDBP00001172	18.0233	2.18E-05	0.001105
ENSG00000135503	TSDBP00001249	20.2002	6.97E-06	0.000464
ENSG00000135480	TSDBP00001253	14.1309	0.000171	0.00502
ENSG00000182628	TSDBP00001298	25.2931	4.92E-07	5.19E-05
ENSG00000168530	TSDBP00001363	21.4285	3.67E-06	0.000274
ENSG00000122482	TSDBP00001420	32.8799	9.8E-09	2.27E-06
ENSG00000140945	TSDBP00001630	29.0585	7.02E-08	9.86E-06
ENSG00000166558	TSDBP00001731	22.0771	2.62E-06	0.000214
ENSG00000103089	TSDBP00001768	15.4106	8.65E-05	0.003074
ENSG00000111886	TSDBP00001860	13.5991	0.000226	0.006379
ENSG00000095261	TSDBP00001866	20.531	5.87E-06	0.000409
ENSG00000136861	TSDBP00001870	16.3132	5.37E-05	0.002147
ENSG00000105376	TSDBP00002035	15.3483	8.94E-05	0.003152
ENSG00000198003	TSDBP00002050	30.9546	2.64E-08	4.8E-06
ENSG00000105397	TSDBP00002060	37.353	9.86E-10	3.98E-07
ENSG00000130816	TSDBP00002065	17.908	2.32E-05	0.001144
ENSG00000188157	TSDBP00002202	14.7011	0.000126	0.004042
ENSG00000070193	TSDBP00002267	19.3154	1.11E-05	0.000647
ENSG00000110104	TSDBP00002310	25.923	3.55E-07	3.87E-05
ENSG00000134824	TSDBP00002325	22.0525	2.65E-06	0.000214
ENSG00000166123	TSDBP00002374	16.3352	5.31E-05	0.002132
ENSG00000188993	TSDBP00002481	18.1137	2.08E-05	0.00106
ENSG00000139350	TSDBP00002522	24.543	7.27E-07	7.02E-05
ENSG00000106571	TSDBP00002615	50.9481	9.48E-13	1.1E-09
ENSG00000152223	TSDBP00002695	16.2827	5.46E-05	0.002173
ENSG00000106100	TSDBP00002722	14.6631	0.000129	0.004075
ENSG00000106125	TSDBP00002736	18.1989	1.99E-05	0.001037
ENSG00000091583	TSDBP00002787	24.9138	6E-07	6.11E-05
ENSG00000075461	TSDBP00002815	13.2136	0.000278	0.007471
ENSG00000007312	TSDBP00002833	15.7305	0.000073	0.002719
ENSG00000108604	TSDBP00002836	91.2726	1.51E-20	4.68E-17
ENSG00000082014	TSDBP00002996	28.4426	9.65E-08	1.28E-05
ENSG00000033050	TSDBP00002997	46.3003	1.01E-11	7.02E-09
ENSG00000081479	TSDBP00003161	25.0178	5.68E-07	5.85E-05
ENSG00000042832	TSDBP00003220	35.9669	2.01E-09	6.9E-07
ENSG00000066827	TSDBP00003227	27.4778	1.59E-07	2.05E-05
ENSG00000143786	TSDBP00003341	13.7442	0.000209	0.005982

ENSG00000174059	TSDBP00003355	15.456	8.44E-05	0.003037
ENSG00000198892	TSDBP00003405	12.6184	0.000382	0.009626
ENSG00000133067	TSDBP00003411	19.5908	9.59E-06	0.000581
ENSG00000188770	TSDBP00003424	14.3832	0.000149	0.004534
ENSG00000263528	TSDBP00003440	15.9309	6.57E-05	0.002539
ENSG00000165813	TSDBP00003504	14.7525	0.000123	0.003961
ENSG00000019549	TSDBP00003546	13.9158	0.000191	0.005552
ENSG00000129195	TSDBP00003549	18.1403	2.05E-05	0.001055
ENSG00000196535	TSDBP00003570	14.3971	0.000148	0.004516
ENSG00000174106	TSDBP00003606	31.561	1.93E-08	3.98E-06
ENSG00000078177	TSDBP00003637	17.5106	2.86E-05	0.00132
ENSG00000077782	TSDBP00003677	26.1337	3.19E-07	3.56E-05
ENSG00000170265	TSDBP00003727	12.5275	0.000401	0.009972
ENSG00000162946	TSDBP00003865	33.2314	8.18E-09	2.11E-06
ENSG00000132510	TSDBP00004089	13.2247	0.000276	0.007463
ENSG00000129245	TSDBP00004113	48.6039	3.13E-12	2.9E-09
ENSG00000173013	TSDBP00004170	15.5547	8.02E-05	0.002932
ENSG00000159214	TSDBP00004230	26.738	2.33E-07	2.77E-05
ENSG00000178028	TSDBP00004233	15.0083	0.000107	0.003589
ENSG00000177125	TSDBP00004259	36.281	1.71E-09	6.1E-07
ENSG00000160447	TSDBP00004280	14.7864	0.00012	0.003932
ENSG00000167130	TSDBP00004286	39.5072	3.27E-10	1.6E-07
ENSG00000170989	TSDBP00004648	16.941	3.86E-05	0.001673
ENSG00000019169	TSDBP00004803	23.1571	1.49E-06	0.000133
ENSG00000074047	TSDBP00004812	16.9861	3.77E-05	0.001649
ENSG00000171557	TSDBP00004832	19.2503	1.15E-05	0.000662
ENSG00000107863	TSDBP00004916	26.757	2.31E-07	2.77E-05
ENSG00000059377	TSDBP00005163	12.6845	0.000369	0.009394
ENSG00000122420	TSDBP00005263	30.7614	2.92E-08	5.11E-06
ENSG00000134242	TSDBP00005371	22.4514	2.16E-06	0.000184
ENSG00000162775	TSDBP00005395	26.1727	3.12E-07	3.53E-05
ENSG00000099991	TSDBP00005563	36.7377	1.35E-09	5.01E-07
ENSG00000102098	TSDBP00005603	24.73632	4.25E-06	0.00031
ENSG00000165959	TSDBP00005833	12.9393	0.000322	0.008377
ENSG00000144218	TSDBP00005868	15.1309	0.0001	0.00342
ENSG00000115446	TSDBP00005903	16.1219	5.94E-05	0.002315
ENSG00000135924	TSDBP00005930	32.85074	7.35E-08	1.02E-05
ENSG00000182871	TSDBP00005965	16.591	4.64E-05	0.001913
ENSG00000104885	TSDBP00006057	20.6201	5.6E-06	0.000398
ENSG00000125912	TSDBP00006064	16.9434	3.85E-05	0.001673
ENSG00000105289	TSDBP00006070	20.3574	6.42E-06	0.000435
ENSG00000167670	TSDBP00006079	12.8362	0.00034	0.008758
ENSG00000180447	TSDBP00006124	31.0917	2.46E-08	4.56E-06
ENSG00000088386	TSDBP00006250	17.0436	3.65E-05	0.001604
ENSG00000188738	TSDBP00006261	16.1304	5.91E-05	0.002313
ENSG00000186806	TSDBP00006294	29.5928	5.33E-08	7.72E-06
ENSG00000167749	TSDBP00006306	13.7699	0.000207	0.005951
ENSG00000161681	TSDBP00006311	98.0687	4.04E-23	3.75E-19
ENSG00000006555	TSDBP00006386	28.7342	8.3E-08	1.13E-05
ENSG00000269058	TSDBP00006414	19.1842	1.19E-05	0.000681
ENSG00000176225	TSDBP00006421	17.5056	2.86E-05	0.00132
ENSG00000138182	TSDBP00006492	14.568	0.000135	0.004221
ENSG00000137673	TSDBP00006579	21.8037	3.02E-06	0.000235
ENSG00000165084	TSDBP00006619	18.7239	1.51E-05	0.000839

ENSG00000116035	TSDBP00006665	13.221	0.000277	0.007463
ENSG00000187605	TSDBP00006687	30.7703	2.9E-08	5.11E-06
ENSG00000115363	TSDBP00006703	15.0492	0.000105	0.003545
ENSG00000163071	TSDBP00006893	19.5678	9.71E-06	0.000585
ENSG00000122435	TSDBP00007014	14.1994	0.000164	0.004872
ENSG00000155886	TSDBP00007065	15.0055	0.000107	0.003589
ENSG00000177728	TSDBP00007300	13.5391	0.000234	0.00653
ENSG00000167895	TSDBP00007323	15.4225	0.000086	0.003074
ENSG00000266074	TSDBP00007349	31.9018	1.62E-08	3.41E-06
ENSG00000169710	TSDBP00007379	18.9487	1.34E-05	0.000758
ENSG00000035862	TSDBP00007420	14.9435	0.000111	0.003696
ENSG00000133195	TSDBP00007486	13.0921	0.000297	0.007813
ENSG00000142197	TSDBP00007512	15.0646	0.000104	0.003529
ENSG00000171161	TSDBP00007564	32.6781	1.09E-08	2.41E-06
ENSG00000124608	TSDBP00007654	13.2766	0.000269	0.007309
ENSG00000011295	TSDBP00007776	17.6063	2.72E-05	0.001287
ENSG00000151364	TSDBP00007966	13.7156	0.000213	0.00605
ENSG00000142173	TSDBP00008090	13.1437	0.000288	0.007644
ENSG00000125812	TSDBP00008135	27.3861	1.67E-07	2.12E-05
ENSG00000172534	TSDBP00008221	14.2128	0.000163	0.004853
ENSG00000089820	TSDBP00008222	32.0095	1.53E-08	3.3E-06
ENSG00000165458	TSDBP00008484	31.3108	2.2E-08	4.44E-06
ENSG00000054967	TSDBP00008494	15.795	7.06E-05	0.002651
ENSG00000062282	TSDBP00008512	14.7677	0.000122	0.003944
ENSG00000101096	TSDBP00008550	38.7849	4.73E-10	2.09E-07
ENSG00000204103	TSDBP00008571	19.7607	8.78E-06	0.000546
ENSG00000182584	TSDBP00008632	27.1149	1.92E-07	2.41E-05
ENSG00000142655	TSDBP00008715	24.882	6.09E-07	6.14E-05
ENSG00000069812	TSDBP00008906	18.3744	1.81E-05	0.00097
ENSG00000150977	TSDBP00008993	17.2452	3.29E-05	0.001488
ENSG00000204856	TSDBP00009018	15.8597	6.82E-05	0.002589
ENSG00000137766	TSDBP00009061	16.3757	5.19E-05	0.002093
ENSG00000128710	TSDBP00009080	44.9159	2.06E-11	1.19E-08
ENSG00000144320	TSDBP00009091	12.9658	0.000317	0.008311
ENSG00000070190	TSDBP00009114	15.8276	6.94E-05	0.002616
ENSG00000164035	TSDBP00009116	15.2586	9.37E-05	0.003267
ENSG00000065485	TSDBP00009158	13.912	0.000192	0.005552
ENSG00000173706	TSDBP00009171	34.5575	4.14E-09	1.29E-06
ENSG00000180263	TSDBP00009228	15.1907	9.72E-05	0.003326
ENSG00000114857	TSDBP00009297	30.6763	3.05E-08	5.19E-06
ENSG00000112701	TSDBP00009344	19.5999	9.55E-06	0.000581
ENSG00000119522	TSDBP00009366	12.8896	0.00033	0.008559
ENSG00000163534	TSDBP00009430	17.7362	2.54E-05	0.001227
ENSG00000165669	TSDBP00009446	16.6771	4.43E-05	0.001859
ENSG00000132970	TSDBP00009456	23.628	1.17E-06	0.000107
ENSG00000171115	TSDBP00009478	19.7656	8.75E-06	0.000546
ENSG00000049759	TSDBP00009585	22.8855	1.72E-06	0.000152
ENSG00000061794	TSDBP00009611	19.4302	1.04E-05	0.000618
ENSG00000030110	TSDBP00009718	15.8553	6.84E-05	0.002589
ENSG00000114805	TSDBP00009802	16.4121	0.000051	0.002065
ENSG00000047457	TSDBP00009825	16.2558	5.53E-05	0.002192
ENSG00000101773	TSDBP00009926	17.9507	2.27E-05	0.001126
ENSG00000141446	TSDBP00009935	21.2293	4.07E-06	0.0003
ENSG00000123064	TSDBP00009962	14.7673	0.000122	0.003944

ENSG00000111344	TSDBP00009963	46.6443	8.51E-12	6.58E-09
ENSG00000188647	TSDBP00009985	19.6615	9.24E-06	0.000567
ENSG00000137090	TSDBP00009991	18.2144	1.97E-05	0.001032
ENSG00000074276	TSDBP00010113	14.8021	0.000119	0.003913
ENSG00000146083	TSDBP00010157	13.5735	0.000229	0.006447
ENSG00000176623	TSDBP00010187	17.5318	2.83E-05	0.00132
ENSG00000007402	TSDBP00010323	34.1008	5.23E-09	1.47E-06
ENSG00000119681	TSDBP00010343	15.5512	8.03E-05	0.002932
ENSG00000100767	TSDBP00010357	21.614	3.33E-06	0.000253
ENSG00000164362	TSDBP00010381	18.4493	1.74E-05	0.000938
ENSG00000134324	TSDBP00010435	19.3093	1.11E-05	0.000647
ENSG00000104237	TSDBP00010491	18.5074	1.69E-05	0.000922
ENSG00000181195	TSDBP00010512	17.6055	2.72E-05	0.001287
ENSG00000120156	TSDBP00010605	14.3449	0.000152	0.004612
ENSG00000183111	TSDBP00010713	19.8536	8.36E-06	0.000531
ENSG00000155846	TSDBP00010714	18.7064	1.52E-05	0.000839
ENSG00000168348	TSDBP00010741	17.3378	3.13E-05	0.001423
ENSG00000170310	TSDBP00010894	38.8554	4.56E-10	2.09E-07
ENSG00000111962	TSDBP00011045	22.6038	1.99E-06	0.000171
ENSG00000163611	TSDBP00011092	62.6067	2.52E-15	5.84E-12
ENSG00000129566	TSDBP00011121	15.5065	8.22E-05	0.00299
ENSG00000165684	TSDBP00011165	12.6407	0.000377	0.009538
ENSG00000197070	TSDBP00011190	17.8591	2.38E-05	0.001162
ENSG00000104881	TSDBP00011439	32.7483	1.05E-08	2.38E-06
ENSG00000079435	TSDBP00011468	12.6495	0.000376	0.009519
ENSG00000011478	TSDBP00011526	24.8113	6.32E-07	6.28E-05
ENSG00000063169	TSDBP00011544	15.0289	0.000106	0.003571
ENSG00000163629	TSDBP00011607	34.4165	4.45E-09	1.29E-06
ENSG00000152591	TSDBP00011612	18.0107	0.000022	0.001109
ENSG00000152583	TSDBP00011635	42.1514	8.45E-11	4.61E-08
ENSG00000145996	TSDBP00011706	20.2673	6.73E-06	0.000452
ENSG00000167971	TSDBP00011736	19.6755	9.18E-06	0.000567
ENSG00000187535	TSDBP00011749	15.3277	9.04E-05	0.003176
ENSG00000251692	TSDBP00011750	14.6693	0.000128	0.004075
ENSG00000102854	TSDBP00011796	22.0087	2.71E-06	0.000217
ENSG00000167074	TSDBP00012005	12.6059	0.000385	0.009664
ENSG00000107447	TSDBP00012289	23.6586	1.15E-06	0.000107
ENSG00000166024	TSDBP00012304	17.8902	2.34E-05	0.001148
ENSG00000165480	TSDBP00012380	21.9076	2.86E-06	0.000227
ENSG00000142156	TSDBP00012551	26.657	2.43E-07	2.85E-05
ENSG00000126759	TSDBP00012604	15.5557	8.01E-05	0.002932
ENSG00000171766	TSDBP00012670	13.182	0.000283	0.007572
ENSG00000110719	TSDBP00012702	16.7008	4.38E-05	0.001846
ENSG00000186310	TSDBP00012748	14.7047	0.000126	0.004042
ENSG00000138152	TSDBP00012921	15.9022	6.67E-05	0.002567
ENSG00000171517	TSDBP00013160	16.478	4.92E-05	0.002001
ENSG00000186174	TSDBP00013279	13.1776	0.000283	0.007572
ENSG00000184949	TSDBP00013357	17.6552	2.65E-05	0.001267
ENSG00000148572	TSDBP00013397	20.8425	4.99E-06	0.000362
ENSG00000132561	TSDBP00013413	46.2085	1.06E-11	7.02E-09
ENSG00000138029	TSDBP00013586	13.7428	0.00021	0.005982
ENSG00000176029	TSDBP00013647	14.9012	0.000113	0.003726
ENSG00000111816	TSDBP00013678	14.5526	0.000136	0.00423
ENSG00000086159	TSDBP00013748	17.1268	0.000035	0.001553

ENSG00000110934	TSDBP00013760	14.1558	0.000168	0.00497
ENSG00000167566	TSDBP00013770	17.1713	3.42E-05	0.001525
ENSG00000167548	TSDBP00013777	33.1673	8.46E-09	2.12E-06
ENSG00000135763	TSDBP00013844	16.9192	0.000039	0.001682
ENSG00000128805	TSDBP00013929	30.4476	3.43E-08	5.58E-06
ENSG00000170324	TSDBP00013930	30.6581	3.08E-08	5.19E-06
ENSG00000134215	TSDBP00013991	19.8728	8.28E-06	0.00053
ENSG00000134186	TSDBP00014003	31.1578	2.38E-08	4.5E-06
ENSG00000157020	TSDBP00014057	14.2229	0.000162	0.004853
ENSG00000164694	TSDBP00014105	32.8977	9.71E-09	2.27E-06
ENSG00000160710	TSDBP00014163	47.4442	5.66E-12	4.77E-09
ENSG00000137764	TSDBP00014181	17.1091	3.53E-05	0.001559
ENSG00000149182	TSDBP00014292	20.0136	7.69E-06	0.000499
ENSG00000019505	TSDBP00014304	12.9351	0.000322	0.008377
ENSG00000149179	TSDBP00014330	21.6611	3.25E-06	0.000249
ENSG00000132952	TSDBP00014357	45.1588	1.82E-11	1.13E-08
ENSG00000107611	TSDBP00014402	16.0361	6.21E-05	0.00241
ENSG00000081791	TSDBP00014431	14.0249	0.00018	0.005295
ENSG00000114654	TSDBP00014495	58.8255	1.72E-14	2.66E-11
ENSG00000104447	TSDBP00014560	15.2329	0.000095	0.0033
ENSG00000105717	TSDBP00014705	30.197	3.9E-08	6.13E-06
ENSG00000187664	TSDBP00014709	26.7484	2.32E-07	2.77E-05
ENSG00000180316	TSDBP00014802	13.5325	0.000234	0.00653
ENSG00000255587	TSDBP00014807	12.8417	0.000339	0.008757
ENSG00000112559	TSDBP00014832	33.8598	5.92E-09	1.61E-06
ENSG00000161911	TSDBP00014898	14.6603	0.000129	0.004075
ENSG00000134917	TSDBP00014960	14.3174	0.000154	0.004665
ENSG00000163492	TSDBP00015004	30.3476	3.61E-08	5.77E-06
ENSG00000141179	TSDBP00015084	13.1676	0.000285	0.007591
ENSG00000161618	TSDBP00015211	13.0993	0.000295	0.007805
ENSG00000063127	TSDBP00015236	25.1041	5.43E-07	5.66E-05
ENSG00000104804	TSDBP00015239	18.133	2.06E-05	0.001055
ENSG00000182264	TSDBP00015243	14.4204	0.000146	0.004481
ENSG00000163380	TSDBP00015277	16.8358	4.08E-05	0.001744
ENSG00000154920	TSDBP00015384	15.888	6.72E-05	0.002575
ENSG00000136169	TSDBP00015539	25.4888	4.45E-07	4.8E-05
ENSG00000123977	TSDBP00015591	15.4169	8.62E-05	0.003074
ENSG00000124203	TSDBP00015630	21.7156	3.16E-06	0.000244
ENSG00000005189	TSDBP00015654	40.2592	2.22E-10	1.14E-07
ENSG00000075391	TSDBP00015789	21.3807	3.77E-06	0.00028
ENSG00000117009	TSDBP00015830	15.7587	0.000072	0.002692
ENSG00000158106	TSDBP00016029	59.7924	1.05E-14	1.95E-11
ENSG00000178209	TSDBP00016041	88.0089	6.52E-21	3.02E-17
ENSG00000169436	TSDBP00016089	54.2713	1.75E-13	2.32E-10
ENSG00000186472	TSDBP00016154	16.6451	4.51E-05	0.001876
ENSG00000118690	TSDBP00016246	14.2152	0.000163	0.004853
ENSG00000079112	TSDBP00016359	17.5501	0.000028	0.001318
ENSG00000198408	TSDBP00016488	13.6036	0.000226	0.006379
ENSG00000148843	TSDBP00016516	34.4931	4.28E-09	1.29E-06
ENSG00000105373	TSDBP00016620	21.5476	3.45E-06	0.00026
ENSG00000079819	TSDBP00016766	14.2468	0.00016	0.004812
ENSG00000068383	TSDBP00016916	16.5685	4.69E-05	0.001925
ENSG00000100099	TSDBP00017013	31.2254	2.3E-08	4.44E-06
ENSG00000204842	TSDBP00017078	50.3707	1.27E-12	1.31E-09

ENSG00000007908	TSDBP00017097	13.6468	0.000221	0.006257
ENSG00000174175	TSDBP00017098	37.4086	9.58E-10	3.98E-07
ENSG00000080345	TSDBP00017276	16.168	0.000058	0.002279
ENSG00000183091	TSDBP00017287	25.9364	3.53E-07	3.87E-05
ENSG00000164808	TSDBP00017473	18.8681	0.000014	0.000787
ENSG00000174738	TSDBP00017481	13.8057	0.000203	0.005857
ENSG00000131016	TSDBP00017500	28.6942	8.48E-08	1.14E-05
ENSG00000136925	TSDBP00017583	14.624	0.000131	0.00414
ENSG00000146909	TSDBP00017653	29.8882	4.58E-08	7.01E-06
ENSG00000109586	TSDBP00017678	20.3592	6.42E-06	0.000435
ENSG00000113360	TSDBP00017690	14.9312	0.000112	0.003706
ENSG00000160183	TSDBP00017901	17.5097	2.86E-05	0.00132
ENSG00000158863	TSDBP00017958	13.7439	0.00021	0.005982
ENSG00000107164	TSDBP00018211	13.3453	0.000259	0.007129
ENSG00000101204	TSDBP00018229	15.7195	7.35E-05	0.002723
ENSG00000101187	TSDBP00018233	18.5555	1.65E-05	0.000905
ENSG00000103544	TSDBP00018494	12.5627	0.000394	0.009837
ENSG00000183690	TSDBP00018535	18.3211	1.87E-05	0.000985
ENSG00000134759	TSDBP00018585	31.2629	2.25E-08	4.44E-06
ENSG00000139767	TSDBP00018626	22.7488	1.85E-06	0.00016
ENSG00000022840	TSDBP00018636	14.575	0.000135	0.00422
ENSG00000110876	TSDBP00018654	13.0301	0.000307	0.008053
ENSG00000135114	TSDBP00018660	26.2509	3E-07	3.43E-05
ENSG00000171435	TSDBP00018682	24.7976	6.37E-07	6.28E-05
ENSG00000103723	TSDBP00018695	22.2485	2.4E-06	0.000201
ENSG00000197299	TSDBP00018703	17.9775	2.24E-05	0.001123
ENSG00000140519	TSDBP00018719	20.1897	7.01E-06	0.000464
ENSG00000169744	TSDBP00018805	13.3037	0.000265	0.007225
ENSG00000136274	TSDBP00018877	13.4487	0.000245	0.006787
ENSG00000015520	TSDBP00018879	16.8411	4.06E-05	0.001743
ENSG00000074755	TSDBP00018964	22.106	2.58E-06	0.000214
ENSG00000196689	TSDBP00018972	14.2896	0.000157	0.004719
ENSG00000170486	TSDBP00019152	26.3616	2.83E-07	3.28E-05
ENSG00000147533	TSDBP00019170	12.5856	0.000389	0.009743
ENSG00000178522	TSDBP00019238	15.2001	9.67E-05	0.003326
ENSG00000173039	TSDBP00019334	13.5334	0.000234	0.00653
ENSG00000172543	TSDBP00019418	14.5166	0.000139	0.004295
ENSG00000164440	TSDBP00019436	20.3989	6.29E-06	0.000435
ENSG00000203734	TSDBP00019445	35.1733	3.02E-09	1E-06
ENSG00000205758	TSDBP00019496	34.4482	4.38E-09	1.29E-06
ENSG00000205927	TSDBP00019510	14.418	0.000146	0.004481
ENSG00000159110	TSDBP00019513	19.9757	7.84E-06	0.000505
ENSG00000205726	TSDBP00019522	17.7422	2.53E-05	0.001227
ENSG00000015133	TSDBP00019750	24.736	6.57E-07	6.41E-05
ENSG00000004399	TSDBP00019829	19.0382	1.28E-05	0.000728
ENSG00000073861	TSDBP00019949	15.2265	9.54E-05	0.003301
ENSG00000002919	TSDBP00019954	20.1373	7.21E-06	0.000474
ENSG00000205639	TSDBP00019960	22.0543	2.65E-06	0.000214
ENSG00000135476	TSDBP00020011	13.9506	0.000188	0.005473
ENSG00000107736	TSDBP00020038	25.3356	4.82E-07	5.14E-05
ENSG00000173578	TSDBP00020070	23.35177	8.5E-06	0.000536
ENSG00000174837	TSDBP00020093	14.4554	0.000144	0.004422
ENSG00000125730	TSDBP00020116	19.2623	1.14E-05	0.000661
ENSG00000134539	TSDBP00020235	17.4303	2.98E-05	0.001368

ENSG00000164236	TSDBP00020293	18.8596	1.41E-05	0.000788
ENSG00000037474	TSDBP00020311	13.2268	0.000276	0.007463
ENSG00000180834	TSDBP00020510	23.716	1.12E-06	0.000105
ENSG00000125503	TSDBP00020625	17.9553	2.26E-05	0.001126
ENSG00000178752	TSDBP00020659	20.6142	5.62E-06	0.000398
ENSG00000130294	TSDBP00020676	19.96207	4.63E-05	0.001913
ENSG00000146830	TSDBP00020808	20.3651	6.4E-06	0.000435
ENSG00000113460	TSDBP00020823	15.4916	8.29E-05	0.003003
ENSG00000084636	TSDBP00020963	33.0255	9.1E-09	2.22E-06
ENSG00000130876	TSDBP00021210	14.9106	0.000113	0.00372
ENSG00000143450	TSDBP00021262	13.4842	0.000241	0.00668
ENSG00000215853	TSDBP00021264	27.6292	1.47E-07	1.92E-05
ENSG00000143390	TSDBP00021277	19.4252	1.05E-05	0.00062
ENSG00000134291	TSDBP00021322	16.7494	4.27E-05	0.001817
ENSG00000106682	TSDBP00021557	24.2691	8.38E-07	7.93E-05
ENSG00000125637	TSDBP00021692	12.6774	0.00037	0.009404
ENSG00000164654	TSDBP00022032	15.1926	9.71E-05	0.003326
ENSG00000121964	TSDBP00022496	20.1133	7.3E-06	0.000477
ENSG00000116350	TSDBP00022799	17.227	3.32E-05	0.001495
ENSG00000145832	TSDBP00022947	15.2853	9.24E-05	0.003234
ENSG00000050628	TSDBP00022967	22.742	1.85E-06	0.00016
ENSG00000145700	TSDBP00023017	13.3108	0.000264	0.007219
ENSG00000245848	TSDBP00023034	16.6563	4.48E-05	0.001872
ENSG00000107731	TSDBP00023157	27.011	2.02E-07	2.5E-05
ENSG00000124209	TSDBP00023337	13.9784	0.000185	0.00541
ENSG00000136929	TSDBP00023403	15.3636	8.87E-05	0.00314
ENSG00000099769	TSDBP00023506	12.8142	0.000344	0.008837
ENSG00000166960	TSDBP00023639	18.4823	1.71E-05	0.000927
ENSG00000196814	TSDBP00023751	14.5923	0.000133	0.004196
ENSG00000101331	TSDBP00023782	29.8409	4.69E-08	7.01E-06
ENSG00000172139	TSDBP00023796	37.0037	1.18E-09	4.56E-07
ENSG00000183137	TSDBP00023874	15.8711	6.78E-05	0.002588
ENSG00000134490	TSDBP00023901	19.4455	1.04E-05	0.000618
ENSG00000184811	TSDBP00024051	29.1424	6.72E-08	9.59E-06
ENSG00000132017	TSDBP00024056	12.7587	0.000354	0.009078
ENSG00000213699	TSDBP00024138	20.5554	5.79E-06	0.000407
ENSG00000160294	TSDBP00024200	15.7136	7.37E-05	0.002723
ENSG00000184925	TSDBP00024214	18.3344	1.85E-05	0.00098
ENSG00000164002	TSDBP00024294	21.8765	2.91E-06	0.000229
ENSG00000168016	TSDBP00024575	14.9222	0.000112	0.003711
ENSG00000138600	TSDBP00024580	23.4032	1.31E-06	0.000119
ENSG00000085552	TSDBP00024588	14.6884	0.000127	0.004056
ENSG00000176601	TSDBP00024645	17.207	3.35E-05	0.001501
ENSG00000138303	TSDBP00024669	18.3443	1.84E-05	0.00098
ENSG00000113597	TSDBP00024688	12.5271	0.000401	0.009972
ENSG00000196074	TSDBP00024710	16.2182	5.64E-05	0.002226
ENSG00000174564	TSDBP00024759	33.3143	7.84E-09	2.08E-06
ENSG00000189001	TSDBP00024771	22.2888	2.35E-06	0.000198
ENSG00000167674	TSDBP00024788	17.6814	2.61E-05	0.001254
ENSG00000127663	TSDBP00024791	13.1529	0.000287	0.007628
